# Computational Aspects of Feedback in Neural Circuits

**DOI:** 10.1371/journal.pcbi.0020165

**Published:** 2007-01-19

**Authors:** Wolfgang Maass, Prashant Joshi, Eduardo D Sontag

**Affiliations:** 1 Institute for Theoretical Computer Science, Technische Universitaet Graz, Graz, Austria; 2 Department of Mathematics, Rutgers, The State University of New Jersey, Piscataway, New Jersey, United States of America; Radboud University, The Netherlands

## Abstract

It has previously been shown that generic cortical microcircuit models can perform complex real-time computations on continuous input streams, provided that these computations can be carried out with a rapidly fading memory. We investigate the computational capability of such circuits in the more realistic case where not only readout neurons, but in addition a few neurons *within* the circuit, have been trained for specific tasks. This is essentially equivalent to the case where the output of trained readout neurons is fed back into the circuit. We show that this new model overcomes the limitation of a rapidly fading memory. In fact, we prove that in the idealized case without noise it can carry out any conceivable digital or analog computation on time-varying inputs. But even with noise, the resulting computational model can perform a large class of biologically relevant real-time computations that require a nonfading memory. We demonstrate these computational implications of feedback both theoretically, and through computer simulations of detailed cortical microcircuit models that are subject to noise and have complex inherent dynamics. We show that the application of simple learning procedures (such as linear regression or perceptron learning) to a few neurons enables such circuits to represent time over behaviorally relevant long time spans, to integrate evidence from incoming spike trains over longer periods of time, and to process new information contained in such spike trains in diverse ways according to the current internal state of the circuit. In particular we show that such generic cortical microcircuits with feedback provide a new model for working memory that is consistent with a large set of biological constraints. Although this article examines primarily the computational role of feedback in circuits of neurons, the mathematical principles on which its analysis is based apply to a variety of dynamical systems. Hence they may also throw new light on the computational role of feedback in other complex biological dynamical systems, such as, for example, genetic regulatory networks.

## Author Summary

Circuits of neurons in the brain have an abundance of feedback connections, both on the level of local microcircuits and on the level of synaptic connections between brain areas. But the functional role of these feedback connections is largely unknown. We present a computational theory that characterizes the gain in computational power that feedback can provide in such circuits. It shows that feedback endows standard models for neural circuits with the capability to emulate arbitrary Turing machines. In fact, with suitable feedback they can simulate any dynamical system, in particular any conceivable analog computer. Under realistic noise conditions, the computational power of these circuits is necessarily reduced. But we demonstrate through computer simulations that feedback also provides a significant gain in computational power for quite detailed models of cortical microcircuits with in vivo–like high levels of noise. In particular it enables generic cortical microcircuits to carry out computations that combine information from working memory and persistent internal states in real time with new information from online input streams.

## Introduction

The neocortex performs a large variety of complex computations in real time. It is conjectured that these computations are carried out by a network of cortical microcircuits, where each microcircuit is a rather stereotypical circuit of neurons within a cortical column. A characteristic property of these circuits and networks is an abundance of feedback connections. But the computational function of these feedback connections is largely unknown. Two lines of research have been engaged to solve this problem. In one approach, which one might call the constructive approach, one builds hypothetical circuits of neurons and shows that (under some conditions on the response behavior of its neurons and synapses) such circuits can perform specific computations. In another research strategy, which one might call the analytical approach, one starts with data-based models for actual cortical microcircuits, and analyses which computational operations such “given” circuits can perform under the assumption that a learning process assigns suitable values to some of their parameters (e.g., synaptic efficacies of readout neurons). An underlying assumption of the analytical approach is that complex recurrent circuits, such as cortical microcircuits, cannot be fully understood in terms of the usually considered properties of their components. Rather, system-level approaches that directly address the dynamics of the resulting recurrent neural circuits are needed to complement the bottom-up analysis. This line of research started with the identification and investigation of so-called canonical microcircuits [1]. Several issues related to cortical microcircuits have also been addressed in the work of Grossberg; see [2] and the references therein. Subsequently it was shown that quite complex real-time computations on spike trains can be carried out by such “given” models for cortical microcircuits ([3–6], see [7] for a review). A fundamental limitation of this approach was that only those computations could be modeled that can be carried out with a fading memory, more precisely only those computations that require integration of information over a timespan of 200 ms to 300 ms (its maximal length depends on the amount of noise in the circuit and the complexity of the input spike trains [8]). In particular, computational tasks that require a representation of elapsed time between salient sensory events or motor actions [9], or an internal representation of expected rewards [10–12], working memory [13], accumulation of sensory evidence for decision making [14], the updating and holding of analog variables such as for example the desired eye position [15], and differential processing of sensory input streams according to attentional or other internal states of the neural system [16] could not be modeled in this way. Previous work on concrete examples of artificial neural networks [17] and cortical microcircuit models [18] had already indicated that these shortcomings of the model might arise only if one assumes that learning affects exclusively the synapses of readout neurons that project the results of computations to other circuits or areas, without giving feedback into the circuit from which they extract information. This scenario is in fact rather unrealistic from a biological perspective, since pyramidal neurons in the cortex typically have in addition to their long projecting axon a large number of axon collaterals that provide feedback to the local circuit [19]. Abundant feedback connections also exist on the network level between different brain areas [20]. We show in this article that if one takes feedback connections from readout neurons (that are trained for specific tasks) into account, generic cortical microcircuit models can solve all of the previously listed computational tasks. In fact, one can demonstrate this also for circuits whose underlying noise levels and models for neurons and synapses are substantially more realistic than those which had previously been considered in models for working memory and related tasks.

We show in the Theoretical Analysis section that the significance of feedback for the computational power of neural circuits and other dynamical systems can be explained on the basis of general principles. Theorem 1 implies that a large class of dynamical systems, in particular systems of differential equations that are commonly used to describe the dynamics of firing activity in neural circuits, gain universal computational capabilities for digital and analog computation as soon as one considers them in combination with feedback. A further mathematical result (Theorem 2) implies that the capability to process online input streams in the light of nonfading (or slowly fading) internal states is preserved in the presence of fairly large levels of internal noise. On the basis of this theoretical foundation, one can explain why the computer models of generic cortical microcircuits, which are considered in the section Applications to Generic Cortical Microcircuit Models, are able to solve the previously mentioned benchmark tasks. These results suggest a new computational model for cortical microcircuits, which includes the capability to process online input streams in diverse ways according to different “instructions” that are implemented through high-dimensional attractors of the underlying dynamical system. The high dimensionality of these attractors results from the fact that only a small fraction of synapses need to be modified for their creation. In comparison with the commonly considered low-dimensional attractors, such high-dimensional attractors have additional attractive properties such as compositionality (the intersection of several of them is in general nonempty) and compatibility with real-time computing on online input streams within the same circuit.

The presentation of theoretical results for abstract circuit models in the Theoretical Analysis section is complemented by mathematical details in the Methods section, under the heading Mathematical Definitions, Details to the Proof of Theorem 1, and Examples, and the heading Mathematical Definitions and Details to the Proof of Theorem 2. Details of the computer simulations of more detailed cortical microcircuit models are discussed in Applications to Generic Cortical Microcircuit Models in the Methods section. A discussion of the results of this paper is given in the Discussion section.

## Results

We consider two types of models for neural circuits.

The first model type is mean field models, such as those defined by Equation 6, which models the dynamics of firing rates of neurons in neural circuits. These models have the advantage that they are theoretically tractable, but they have the disadvantage that they do not reflect many known details of cortical microcircuits. However we show that the theoretical results that are proven in the section Theoretical Analysis hold for fairly large classes of dynamical systems. Hence, they potentially also hold for some more detailed models of neural circuits.

The second model type involves quite detailed models of cortical microcircuits consisting of spiking neurons (see the description in Applications to Generic Cortical Microcircuit Models and in Details of the Cortical Microcircuit Models). At present these models cannot be analyzed directly by theoretical methods, hence we can only present statistical data from computer simulations. Our simulation results show that feedback has in these more detailed models a variety of computational consequences that we have derived analytically for the simpler models in Theoretical Analysis. This is not totally surprising insofar as the computations that we consider in the more detailed models can be approximately described in terms of time-varying firing rates for individual neurons.

In both types of models we focus on computations that transform time-varying input streams into time-varying output streams. The input streams are modeled in Theoretical Analysis by time-varying analog functions *u*(*t*) (that might for example represent time-varying firing rates of neurons that provide afferent inputs) and in Applications to Generic Cortical Microcircuit Models by spike trains generated by Poisson processes with time-varying rates. Output streams are analogously modeled by time-varying firing rates, or directly by spike trains. We believe that such online computations, which transform time-varying inputs into time-varying outputs, provide a better framework for modeling cortical processing of information than computations that transform a static vector of numbers (i.e., a batch input) into a static output. Mappings from time-varying inputs to time-varying outputs are referred to as filters (or operators) in mathematics and engineering. A frequently discussed reference class of linear and nonlinear filters includes those that can be described by Volterra or Wiener series (see, e.g., [21]). These filters can equivalently be characterized as those filters that are time-invariant (i.e., they are input-driven and have no “internal clock”) and have a fading memory (see [5]). Fading memory (which is formally defined in Fading-Memory Filters means intuitively that the influence of any specific segment of the input stream on later parts of the output stream becomes negligible when the length of the intervening time interval is sufficiently large. We show in the next two subsections that feedback endows a circuit, which by itself can only carry out computations with fading memory, with flexible ways of combining fading-memory computations on time-varying inputs with computational operations on selected pieces of information in a nonfading memory.

### Theoretical Analysis

The dynamics of firing rates in recurrent circuits of neurons is commonly modeled by systems of nonlinear differential equations of the form

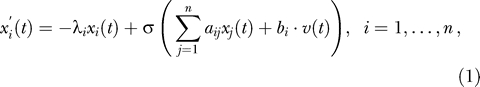
or

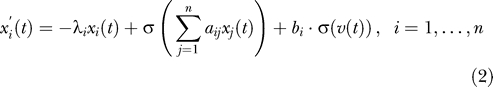
[22–25]. Here each *x_i_*,*i* = 1,…,*n*, is a real-valued variable that represents the current firing rate of the *i^th^* neuron or population of neurons in a recurrent neural circuit, and *v*(*t*) is an external input stream. The coefficients *a_ij_*,*b_i_* denote the strengths of synaptic connections, and *λ_i_* > 0 denotes time constants. The function *σ* is some sigmoidal activation function (nondecreasing, with bounded range). In most models of neural circuits, the parameters are chosen so that the resulting dynamical system has a fading memory for preceding inputs. If one makes the synaptic connection strengths *a_ij_* in Equation 1 or Equation 2 so large that recurrent activity does not dissipate, the neural circuit tends to exhibit persistent memory. But it is usually quite difficult to control the content of this persistent memory, since it tends to be swamped with minor details of external inputs (or initial conditions) from the distant past. Hence this chaotic regime of recurrent neural circuits (see [62] for a review) is apparently also not suitable for biologically realistic online computations that combine new information from the current input with selected (e.g., behaviorally relevant) aspects of external or internal inputs from the past.

Recurrent circuits of neurons (e.g., those described by Equations 1 or 2) are from a mathematical perspective special cases of dynamical systems. The subsequent mathematical results show that a large variety of dynamical systems, in particular also fading-memory systems of type Equation 1 or Equation 2, can overcome in the presence of feedback the computational limitations of a fading memory without necessarily falling into the chaotic regime. In fact, feedback endows them with *universal* capabilities for *analog computing,* in a sense that can be made precise in the following way (see Figure 1A–1C for an illustration):

**Figure 1:**
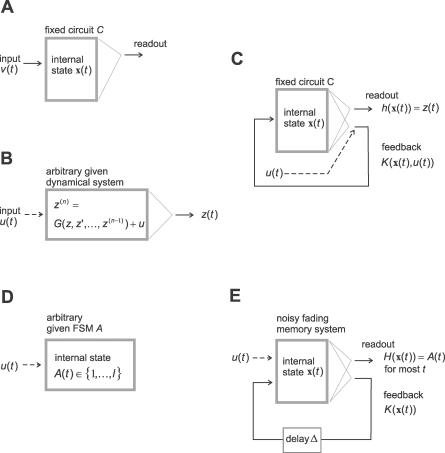
Computational Architectures Considered in Theorems 1 and 2 (A) A fixed circuit *C* whose dynamics is described by the system (Equation 3). (B) An arbitrary given *n^th^* order dynamical system (Equation 4) with external input *u*(*t*). (C) If the input *v*(*t*) to circuit *C* is replaced by a suitable feedback *K*(**x**(*t*),*u*(*t*)), then this fixed circuit *C* can simulate the dynamic response *z*(*t*) of the arbitrarily given system shown in B, for any input stream *u*(*t*). (D) Arbitrary given FSM *A* with l state. (E) A noisy fading-memory system with feedback can reliably reproduce the current state *A*(*t*) of the given FSM *A,* except for timepoints *t* shortly after *A* has switched its state.

#### Theorem 1.

*A large class S_n_ of systems of differential equations of the form*

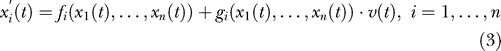

*are in the following sense universal for analog computing:*

*This system (3) can respond to an external input u*(*t*) *with the dynamics of any n^t^ order differential equation of the form*

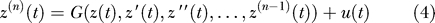

*(for arbitrary smooth functions G*: ℜ*^n^* → ℜ) *if the input term v*(*t*) *is replaced in Equation 3 by a suitable memoryless feedback function K*(*x*_1_(*t*), … ,*x_n_*(*t*),*u*(*t*))*, and if a suitable memoryless readout function h*(**x**(*t*)) *is applied to its internal state* **x**(*t*) = 〈*x*_1_(*t*),…,*x_n_*(*t*)〉*: one can achieve then that h*(**x**(*t*)) = *z*(*t*) *for any solution z*(*t*) *of Equation 4.*

*Also the dynamic responses of all systems consisting of several higher order differential equations of the form Equation 4 can be simulated by fixed systems of the form Equation 3 with a corresponding number of feedbacks.*

This result says more precisely that for any *n^th^* order differential equation (Equation 4) there exists a (memory-free) feedback function K: ℜ*^n^* × ℜ → ℜ and a memory-free readout function *h*: ℜ*^n^* → ℜ (which can both be chosen to be smooth, in particular continuous) so that, for every external input *u*(*t*),*t* ≥ 0, and each solution *z*(*t*) of the forced system (Equation 4), there is an input *u*_0_(*t*) with *u*_0_(*t*) ≡ 0 for all *t* ≥ 1, so that the solution **x**(*t*) = 〈*x*_1_(*t*),…,*x_n_*(*t*)〉 of the fixed system (Equation 3)

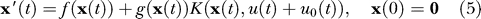
(for *f*: ℜ*^n^* → ℜ*^n^* consisting of 〈*f*_1_,…,*f_n_*〉 and g: ℜ*^n^* → ℜ*^n^* consisting of 〈*g*_1_,…*g*_n_〉) is such that

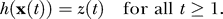
Note that the function *u*_0_(*t*), which is added to the input for *t* < 1 (whereas *u*_0_(*t*) = 0 for *t* ≥ 1), allows the system (Equation 3) (and Equation 5) to simulate with a standardized initial condition **x**(0) = **0** for any solution of Equation 4 with arbitrary initial conditions.

Theorem 1 implies that even if some fixed dynamical system (Equation 3) from the class *S_n_* has fading memory, a suitable feedback *K* and readout function *h* will enable it to carry out specific computations with persistent memory. In fact, it can carry out *any* computation with persistent memory that could possibly be carried out by *any* dynamical system (Equation 4). To get a clear understanding of this universality property, one should note that the feedback function *K* and the readout function *h* depend only on the function *G* that characterizes the simulated system (Equation 4), but not on the external input *u*(*t*) or the particular solution *z*(*t*) of Equation 4 that it simulates. Hence, Theorem 1 implies in particular that any system (Equation 3) that belongs to the class *S_n_* has in conjunction with several feedbacks the computational power of a universal Turing machine (see [26] or [27] for relevant concepts from computation theory). This follows from the fact that every Turing machine (hence any conceivable digital computation, most of which require a persistent memory) can be simulated by systems of equations of the form Equation 4 (this was shown in [28] for the case with continuous time, and in [29,30] for recurrent neural networks with discrete time; see [31] for a review). But possibly more relevant for applications to biological systems is the fact that any fixed system (Equation 3) that belongs to the class *S_n_* is able to emulate any conceivable *continuous* dynamic response to an input stream *u*(*t*) if it receives a suitable feedback *K*(**x**(*t*),*u*(*t*)), where *K* can always be chosen to be continuous. Hence one may argue that these systems (Equation 3) are also universal for *analog* computing on time-varying inputs.

The class *S_n_* of dynamical systems become through feedback universal for analog computing subsumes systems of the form

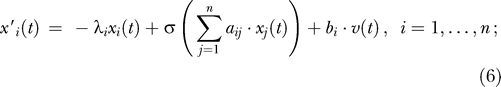
for example, if the *λ_i_* are pairwise different and *a_ij_* = 0 for all *i*,*j,* and all *b_i_* are nonzero. Fewer restrictions are needed if more than one feedback to the system (Equation 6) can be used. Systems of the form Equation 1 or Equation 2 are of a slightly different form, since there the activation function *σ* (that has a bounded range) is applied to the term *v*(*t*). But such systems (Equations 1 and 2) can still be universal for all *bounded* analog responses of arbitrary dynamical systems (Equation 4), which are arguably the only ones of interest in a biological context. This follows from the fact that if the external input *u*(*t*) of the system (Equation 4), as well as the resulting solution *z*(*t*) and its derivatives *z* ^(*i*)^ *t* for *i* ≤ *n* − 1, stay within some bounded range, then the values of the feedback *v*(*t*) that is needed for the simulation of Equation 4 by Equation 3 will also stay within a bounded range. More precisely, one has that:

*For each constant c* > 0 *there is a constant C* > 0 *such that: for every external input u*(*t*),*t ≥* 0, *and each solution z*(*t*) *of the forced system (Equation 4) such that*

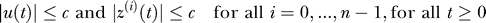

*the input u*_0_ *can be picked so that the feedback*

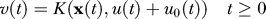

*to Equation 1 or 2 satisfies:*

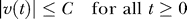



Thus, if we know a priori that we will only deal with solutions of the differential Equation 4 that are bounded by *c*, and inputs are similarly bounded, we could also consider instead of Equation 3 a system such as **x′**(*t*) = *f*(**x**(*t*)) + *g*(**x**(*t*))*σ*(*v*(*t*)) with *f*,*g*: ℜ*^n^* → ℜ*^n^*, where some bounded activation function *σ*: ℜ → ℜ (e.g., *q* · tanh(*v*), for a suitable constant *q*) is applied to the term *v*(*t*) (as in Equation 2). The resulting feedback term *σ*(*K*(**x**(*t*),*u*(*t*) + *u*_0_(*t*))) is then of a mathematical form which is adequate for modeling feedback in neural circuits.

The **proof** of Theorem 1 builds on results from control theory. One important technique in nonlinear control is *feedback linearization* ([32,33]). With this technique, a large class of nonlinear dynamical systems can be transformed through suitable feedback into a linear system (which is then much easier to control). It should be pointed out that this feedback linearization is not a standard linearization method that only yields approximation results, but a method that yields an exact transformation. More generally, one can show in various cases that two dynamical systems, *D*_1_ and *D*_2_, are *feedback equivalent*. The notion of “feedback equivalence” (see Definition of Feedback Equivalence), which is in fact an equivalence relation, expresses that two systems of differential equations can be transformed into each other through application of a suitable feedback and a change of basis in the state space. Such change of basis can be achieved through readout functions *h*(**x**(*t*)) as considered in the claim of Theorem 1. Thus, to show that a fixed system *D*_1_ has the universality property that is specified in the claim of Theorem 1, it suffices to show that *D*_1_ is feedback equivalent to all systems of the form Equation 4. Known results about feedback linearization (see [33], Lemma 5.3.5) imply that the following linear system (Equation 7) is an example of a system *D*_1_ (consisting of *n* differential equations) which has this universality property:

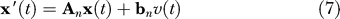
with

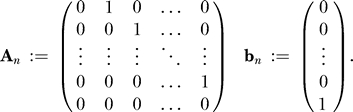
It is in fact very easy to see that any system (Equation 4) can be transformed into the system of Equation 7 with the help of feedback: set *x*_1_(*t*) = *z*(*t*),*x_i_*_+1_(*t*) = *z* ^(*i*)^ for *i* = 1,…,*n* − 1, and use the feedback *v*(*t*) = *G*(**x**(*t*)) + *u*(*t*) in Equation 7. To prove that many other dynamical systems have the same universality property as this system (Equation 7), it suffices to observe that feedback equivalence preserves this universality property.

We define the class *S_n_* in the claim of Theorem 1 as the class of *feedback linearizable* systems, that is, the class of dynamical systems (Equation 3) that are feedback equivalent to some generic linear system. It can be proved (see Lemma in the section Definition of the Class *S_n_*) that every feedback linearizable system (Equation 3) is also feedback equivalent to Equation 7, and hence has the same universality property as Equation 7.

We give in Definition of Class *S_n_* a precise definition of the class *S_n_* in terms of feedback equivalence (which is formally defined in Definition of Feedback Equivalence). We present in Details of the Proof of Theorem 1 a formal proof of the simulation result that is claimed in Theorem 1 (taking also initial conditions into account). In addition we formulate in the section A Characterization of *S_n_* via Lie Brackets an equivalent criterion for a system (Equation 3) to belong to the class *S_n_,* which can be more easily tested for concrete cases of dynamical systems. This criterion makes use of the Lie bracket formalism that is briefly reviewed in Lie Brackets. Applications of this criterion to neural network equations are discussed in Applications to Neural Network Equations. In particular, we use this criterion to show that some dynamical systems (Equation 6) that are defined by standard equations for recurrent neural circuits belong to the class *S_n_*. We also show in Applications to Neural Network Equations that not all systems of the form (Equation 6) belong to the class *S_n_,* rather it depends on the particular choice of parameters *a_ij_* and *b_i_* in Equation 6.

Theorem 1 implies that a generic neural circuit may become through feedback a universal computational device, which cannot only simulate any Turing machine, but also any conceivable model for analog computing with bounded dynamic responses. The “program” of such an arbitrary simulated computing machine gets encapsulated in the static functions *K* that characterize the memoryless computational operations that are required from feedback units, and the static readout functions *h*. Since these functions are static, i.e., time-invariant, and continuous, they provide suitable targets for *learning*. More precisely, to train a generic neural circuit to simulate the dynamic response of an arbitrary dynamical system, it suffices to train—apart from readout neurons—a few neurons within the circuit (or within some external loop) to transform the vector **x**(*t*), which represents the current firing activity of its neurons, and the current external input *u*(*t*) into a suitable feedback value *K*(**x**(*t*),*u*(*t*)). This could, for example, be carried out by training a suitable feedforward neural network within the larger circuit, which can approximate any continuous feedback function *K* [34]. Furthermore, we will show in Applications to Generic Cortical Microcircuit Models that these feedback functions *K* can in many biologically relevant cases be chosen to be linear, so that it would in principle suffice to train a single neuron to compute *K*.

It is known that the memory capacity of such a circuit is reduced to some finite number of bits if these feedback functions *K* are not learnt perfectly, or if there are other sources of noise in the system. More generally, no analog circuit with noise can simulate arbitrary Turing machines [35]. But the subsequent Theorem 2 shows that fading-memory systems with noise and imperfect feedback can still achieve the maximal possible computational power within this a priori limitation: they can simulate any given finite state machine (FSM). Note that any Turing machine with tapes of finite length is a special case of a FSM. Furthermore, any existing digital computer is an FSM, hence the computational capability of FSMs is actually quite large.

To avoid the cumbersome mathematical difficulties that arise when one analyses differential equations with noise, we formulate and prove Theorem 2 on a more abstract level, resorting to the notion of fading-memory filters with noise (see Mathematical Definitions and Details to the Proof of Theorem 2). We assume here that the input–output behavior of those dynamical systems with noise, for which we want to determine the computational impact of (imprecise) state feedback, can be modeled by fading-memory filters with additive noise on their output. The assumption that the amplitude of this noise is bounded is a necessary assumption according to [36]. We refer to [4,5,37] for further discussions of the relationship between models for neural circuits and fading-memory filters. In particular it was shown in [37] that every time-invariant fading-memory filter can be approximated by models for neural circuits, provided that these models reflect the empirically found diversity of time constants of neurons and synapses.

*Theorem 2.* *Feedback allows linear and nonlinear fading-memory systems, even in the presence of additive noise with bounded amplitude, to employ for real-time processing of time-varying inputs the computational capability and nonfading states of any given FSM (see Figure 1D–1E).*

A precise formalization of this result is formulated as Theorem 5 in Precise Statement of Theorem 2, and a formal proof of Theorem 5 is given in Proof of the Precise Statement of Theorem 2. The external input *u*(*t*) can in this case be injected directly into the fading-memory system, so that the feedback *K*(**x**(*t*)) depends only on the internal state **x**(*t*) (see Figure 1E). One essential ingredient of the proof is a method for making sure that noise does not get amplified through feedback: the functions *K* that provide feedback values *K*(**x**(*t*)) can be chosen in such a way that they cancel the impact of imprecision in the values *K*(**x**(*s*)) for immediately preceding time steps *s* < *t*.

### Applications to Generic Cortical Microcircuit Models

We examine in this section computational aspects of feedback in recurrent circuits of spiking neurons that are based on data from cortical microcircuits. The dynamics of these circuits is substantially more complex than the dynamics of circuits described by Equation 6, since it is based on action potentials (spikes) rather than on firing rates. Hence one can expect at best that the temporal dynamics of firing rates in these circuits of spiking neuron is qualitatively similar to that of circuits described by Equation 6.

The preceding theoretical results imply that it is possible for dynamical systems to carry out computations with persistent memory without acquiring all the computational disadvantages of the chaotic regime, where the memory capacity of the system is dominated by noise. Feedback units can create selective “loopholes” into the fading-memory dynamics of a dissipative system that can only be activated by specific patterns in the input or circuit dynamics. In this way the potential content of persistent memory can be controlled by feedback units that have been trained to recognize such patterns. This feedback may arise from a few neurons within the circuit, or from neurons within a larger feedback loop. The task to approximate a suitable feedback function *K* is less difficult than it may appear on first sight, since it suffices in many cases to approximate a *linear* feedback function. The reason is that sufficiently large generic cortical microcircuit models have an inherent kernel property [8], in the sense of machine learning [38]. This means that a large reservoir of diverse nonlinear responses to current and recent input patterns is automatically produced within the recurrent circuit. In particular, nonlinear combinations of variables *a*,*b*,*c*,… (that may result from the circuit input or internal activity) are automatically computed at internal nodes of the circuit. Consequently, numerous low-degree polynomials in these variables *a*,*b*,*c*,… can be approximated by *linear* combinations of outputs of neurons from the recurrent circuit. An example of this effect is demonstrated in Figure 2G, where it is shown that the product of firing rates *r*_3_(*t*) and *r*_4_(*t*) and of two independently varying afferent spike train inputs can be approximated quite well by a linear readout neuron. The kernel property of biologically realistic cortical microcircuit models is apparently supported by the fact that these circuits have many additional nonlinearities in addition to those that appear in Equations 1, 2, and 6.

**Figure 2:**
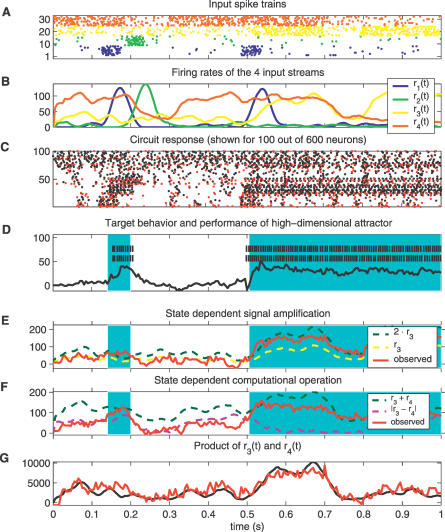
State-Dependent Real-Time Processing of Four Independent Input Streams in a Generic Cortical Microcircuit Model (A) Four input streams, each consisting of eight spike trains generated by Poisson processes with randomly varying rates *r_i_*(*t*),*i* = 1,…4, rates plotted in (B); all rates are given in Hz. The four input streams and the feedback were injected into disjoint sets of neurons in the circuit. (C) Resulting firing activity of 100 out of the 600 I&F neurons in the circuit. Spikes from inhibitory neurons marked in red. (D) Target activation times of the high-dimensional attractor (blue shading), spike trains of two of the eight I&F neurons that were trained to create the high-dimensional attractor by sending their output spike trains back into the circuit, and average firing rate of all eight neurons (lower trace). (E,F) Performance of linear readouts that were trained to switch their real-time computation task depending on the current state of the high-dimensional attractor: output 2 · *r*_3_(*t*) instead of *r*_3_(*t*) if the high-dimensional attractor is on (E), output *r*_3_(*t*) + *r*_4_(*t*) instead of | *r*_3_(*t*) – *r*_4_(*t*) | if the high-dimensional attractor is on (F). (G) Performance of linear readout that was trained to output *r*_3_(*t*) · *r*_4_(*t*), showing that another linear readout from the same circuit can simultaneously carry out nonlinear computations that are invariant to the current state of the high-dimensional attractor.

One formal difference between neurons in the mean field model (Equation 6) and more realistic models for spiking neurons is that the input to a neuron of the latter type consists of postsynaptic potentials, rather than of firing rates. Hence the time-varying input **x**(*t*) to a readout neuron is in this section not a vector of time-varying firing rates, but a smoothed version of the spike trains of all presynaptic neurons. This smoothing is achieved through application of a linear filter with an exponentially decaying kernel, whose time constant of 30 ms models time constants of receptors and postsynaptic membrane of a readout neuron in a qualitative fashion. Thus, if **w** is a vector of synaptic weights, then **w** · **x**(*t*) models the impact of the firing activity of presynaptic neurons on the membrane potential of a readout neuron.

We refer in the following to those neurons where the weights of synaptic connections from neurons within the circuit are adapted for a specific computational task (rather than chosen randomly from distributions that are based on biological data, as for all other synapses in the circuit) as *readout neurons*. The output of a readout neuron was modeled in most of our simulations simply by a weighted sum **w** · **x**(*t*) of the previously described vector **x**(*t*). Such output can be interpreted as the time-varying firing rate of a readout neuron. However, we show in Figure 2 that these readout neurons can (with a moderate loss in performance) also be modeled by spiking neurons, exactly like the other neurons in the simulated circuit. This demonstrates that not only those circuits that receive feedback from external readout neurons, but also generic recurrent circuits in which a few neurons have been trained for a specific task, acquire computational capabilities for real-time processing that are not restricted to computations with fading memory.

Theorem 2 suggests that the training of a few of its neurons enables generic neural circuits to employ persistent internal states for state-dependent processing of online input streams. Previous models for nonfading memory in neural circuits [13,39–41] proposed that it is implemented through low-dimensional attractors in the circuit dynamics. These attractors tend to freeze or to entrain the whole state of the circuit, and thereby shut it off from the online input stream (although independent local attractors could emerge in local subcircuits under some conditions [40]). In contrast, the generation of nonfading memory through a few trained neurons does not entail that the dynamics of the circuit be dominated by their persistent memory states. For example, when a readout neuron gives during some time interval a constant feedback *K*(**x**(*t*)) = *c,* this only constrains the circuit state **x**(*t*) to remain in the sub-manifold {**x:** *K*(**x**) = *c*} of its high-dimensional state space. This sub-manifold is in general high-dimensional. In particular, if *K*(**x**) is a linear function w · x, which often suffices as we will show; the dimensionality of the sub-manifold {**x:** *K*(**x**) = *c*} differs from the dimension of the full state space only by 1. Hence several such sub-manifolds have in general a high-dimensional intersection, and their intersection still leaves sufficiently many degrees of freedom for the circuit state **x**(*t*) to also absorb continuously new information from online input streams. These sub-manifolds are in general not attractors in a strict mathematical sense. Rather, their effective attraction property (or noise-robustness) results from the subsequently described training process (“teacher forcing”). This training process produces weights **w** which have the property that the resulting feedback 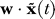 moves on a trajectory of circuit states that goes through states **x~**(*t*) in the neighborhood of the sub-manifold {**x:** *K*(**x**) = *c*}, closer to this sub-manifold.

We simulated generic cortical microcircuit models consisting of 600 integrate-and-fire (I&F) neurons (for Figures 2 and 3), and circuits consisting of 600 Hodgkin–Huxley (HH) neurons (for Figure 4), in either case with a rather high level of noise that reflects experimental data on the high conductance state in vivo [42]. These circuits were not constructed for any particular computational task. In particular, sparse synaptic connectivity between neurons was generated (with a biologically realistic bias towards short connections) by a probabilistic rule. Synaptic parameters were chosen randomly from distributions that depend on the type of pre- and postsynaptic neurons (in accordance with empirical data from [43,44]). More precisely, we used biologically realistic models for dynamic synapses whose individual mixture of paired-pulse depression and facilitation (depending on the type of pre- and postsynaptic neuron) was based on these data. It has previously been shown in [6,8] that the presence of such dynamic synapses extends the timespan of the inherent fading memory of the circuit. However the computational tasks that are considered in this paper require, apart from a nonfading memory, only a fading memory with a rather short timespan (to make the estimation of the current firing rate of input spike trains feasible). Therefore, the biologically more realistic dynamic synapses could be replaced in this model by simple static synapses, without a change in the performance of the circuit for the subsequently considered tasks. All details of the simulated microcircuit models can be found in Details of the Cortical Microcircuit Models. Details of the subsequently discussed computer experiments are given in the sections Technical Details of Figure 5, Technical Details of Figure 2, and Technical Details of Figure 3.

**Figure 3:**
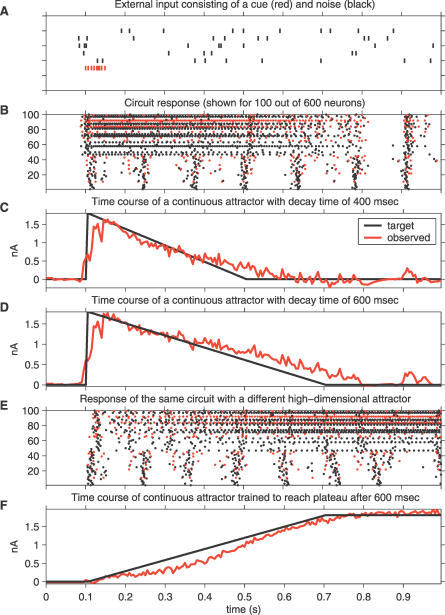
Representation of Time for Behaviorally Relevant Timespans in a Generic Cortical Microcircuit Model (A) Afferent circuit input, consisting of a cue in one channel (red) and random spikes (freshly drawn for each trial) in the other channels. (B) Response of 100 neurons from the same circuit as in Figure 2, which has here two coexisting high-dimensional attractors. The autonomously generated periodic bursts with a periodic frequency of about 8 Hz are not related to the task, and readouts were trained to become invariant to them. (C,D) Feedback from two linear readouts that were simultaneously trained to create and control two high-dimensional attractors. One of them was trained to decay in 400 ms (C), and the other in 600 ms (D) (scale in nA is the average current injected by feedback into a randomly chosen subset of neurons in the circuit). (E) Response of the same neurons as in (B), for the same circuit input, but with feedback from a different linear readout that was trained to create a high-dimensional attractor that increases its activity and reaches a plateau of 600 ms after the occurrence of the cue in the input stream. (F) Feedback from the linear readout that creates this continuous high-dimensional attractor.

**Figure 4:**
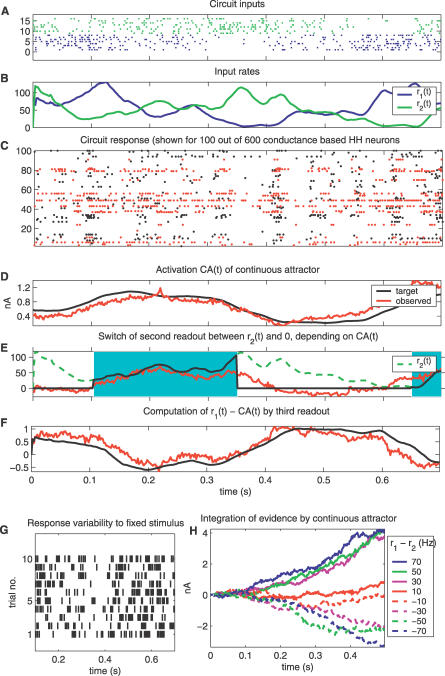
A Model for Analog Real-Time Computation on External and Internal Variables in a Generic Cortical Microcircuit (Consisting of 600 Conductance-Based HH Neurons) (A,B) Two input streams as in Figure 2. (B) Their firing rates *r*_1_(*t*),*r*_2_(*t*). (C) Resulting firing activity of 100 neurons in the circuit. (D) Performance of a neural integrator, generated by feedback from a linear readout that was trained to output at any time *t* an approximation *CA*(*t*) of the integral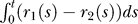 over the difference of both input rates. Feedback values were injected as input currents into a randomly chosen subset of neurons in the circuit. Scale in nA shows average strength of feedback currents, also in (H). (E) Performance of linear readout that was trained to output 0 as long as *CA*(*t*) stayed below 0.83 nA, and to output *r*_2_(*t*) once *CA*(*t*) had crossed this threshold, as long as *CA*(*t*) stayed above 0.66 nA (i.e., in this test run during the shaded time periods). (F) Performance of linear readout trained to output *r*_1_(*t*) − *CA*(*t*), i.e., a combination of external and internal variables, at any time *t* (both *r*_1_ and *CA* normalized into the range [0,1]). (G) Response of a randomly chosen neuron in the circuit for ten repetitions of the same experiment (with input spike trains generated by Poisson processes with the same time course of firing rates), showing biologically realistic trial-to-trial variability. (H) Activity traces of a continuous attractor as in (D), but in eight different trials for eight different fixed values of *r*_1_ and *r*_2_ (shown on the right).

**Figure 5:**
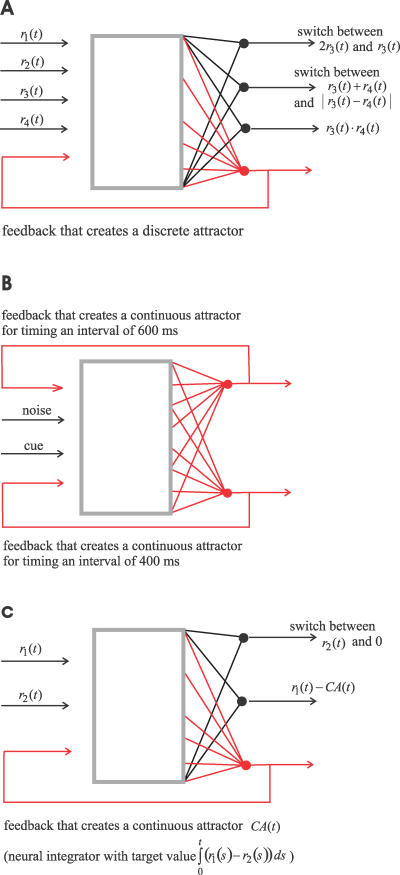
Organization of Input and Output Streams for the Three Computational Tasks Considered in the Computer Simulations Each input stream consisted of multiple spike trains that provided synaptic inputs to individually chosen subsets of neurons in the recurrent circuit (which is indicated by a gray rectangle). (A,C) Input streams consisted of multiple Poisson spike trains with a time-varying firing rate *r*_i_(*t*). (B) Input consisted of a burst (“cue”) in one spike train (which marks the beginning of a time interval) and independent Poisson spike train (“noise”) in the other input channels. (A–C) The actual outputs of the readouts (that were trained individually for each computational task) is shown in Figures 2–4.

We tested three different types of computational tasks for generic neural circuits with feedback. The same neural circuit can be used for each task, only the organization of input and output streams needs to be chosen individually (see Figure 5). The following procedure was applied to train readout neurons, i.e., to adjust the weights of synaptic connections from neurons in the circuit to readout neurons for specific computational tasks (while leaving all other parameters of the generic microcircuit model unchanged): 1) first those readout neurons were trained that provide feedback, then the other readout neurons; 2) during the training of readout neurons that provide feedback, their actual feedback was replaced by a *noisy* version of their target output (“teacher forcing”); 3) each readout neuron was trained by linear regression to output at any time *t* a particular target value *f*(*t*). Linear regression was applied to a set of datapoints of the form 〈**x**(*t*),*f*(*t*)〉 for many timepoints *t,* where **x**(*t*) is a smoothed version of the spike trains of presynaptic neurons (as defined before).

Note that teacher forcing, with noisy versions of target feedback values, trains these readouts to correct errors resulting from imprecision in their preceding feedback (rather than amplifying errors). This training procedure is responsible for the robustness of the dynamics of the resulting closed-loop circuits, in particular for the “attractor” properties of the effectively resulting high-dimensional attractors.

In our first computer experiment, readout neurons were trained to turn a high-dimensional attractor on or off (Figure 2D), in response to bursts in two of the four independent input spike trains. More precisely, eight neurons were trained to represent in their firing activity at any time the information: in which of the input streams, 1 or 2, had a burst most recently occurred? If it had occurred most recently in stream 1, they were trained to fire at 40 Hz, and if a burst had occurred most recently in input stream 2, they were trained not to fire. Hence these neurons were required to represent the nonfading state of a simple FSM, demonstrating in an example the computational capabilities predicted by Theorem 2. Figure 2G demonstrates that the circuit retains its kernel property in spite of the feedback injected into the circuit by these readouts. But beyond the emulation of a simple FSM, the resulting generic cortical microcircuit is able to combine information stored in the current state of the FSM with new information from the online circuit input. For example, Figure 2E shows that other readouts from the same circuit can be trained to amplify their response to specific inputs if the high-dimensional attractor is in the “on” state. Readouts can also be trained to change the function that they compute if the high-dimensional attractor is in the on state (Figure 2F). This provides an example for an online reconfigurable circuit. The readout neurons that provide feedback had been modeled in this computer simulation like the other neurons in the circuit: by I&F neurons with in vivo–like background noise. Hence they can be viewed equivalently as neurons *within* an otherwise generic circuit.

Another difficult problem in computational neuroscience is to explain how neural circuits can implement a parametric memory, i.e., how they can hold and update an *analog* value that may represent, for example, an intended eye position that a neural integrator computes from a sequence of eye-movement commands [45], an estimate of elapsed time [9], or accumulated sensory evidence [14]. Various designs have been proposed for parametric memory in recurrent circuits, where continuous attractors (also referred to as line attractors) hold and update an analog value. But these approaches are inherently brittle [41], and have problems in dealing with high noise or online circuit inputs. On the other hand, Figure 3 shows that dedicated circuit constructions are not necessary, since feedback from readout neurons in *generic* cortical microcircuits models can also create high-dimensional attractors that hold and update an *analog* value for behaviorally relevant timespans. In fact, due to the high-dimensional character of the resulting high-dimensional attractors, two such analog values can be stored and updated independently (Figure 3C and 3D), even within a fairly small circuit. In this example, the readouts that provide feedback were simply trained to increase or reduce their feedback at each timepoint. Note that the resulting circuit activity is qualitatively consistent with recordings from neurons in cortex and striatum during reward expectation [10–12]. A similar ramp-like rise and fall of activity as shown in Figure 3C, 3D, and 3F has also been recorded in neurons of posterior parietal cortex of the macaque in experiments where the monkey had been trained to classify the duration of elapsed time [9]. The high dimensionality of the continuous attractors in this model makes it feasible to constrain the circuit state to stay simultaneously in more than one continuous attractor, thereby making it in principle possible to encode complex movement plans that require specific temporal relationships between individual motor commands.

Our model for parametric memory in cortical circuits is consistent with high noise: Figure 4G shows the typical trial-to-trial variability of a neuron in our simulated circuit of HH neurons with in vivo–like background noise. It qualitatively matches the “wide diversity of neural firing drift patterns in individual fish at all states of tuning” that was observed in the horizontal occulomotor neural integrator in goldfish [15], and the large trial-to-trial variability of neurons in prefrontal cortex of monkeys reported in [10]. In addition, this model is consistent with the surprising plasticity that has been observed even in quite specialized neural integrators [15], since continuous attractors can be created or modified in this model by changing just a few synaptic weights of neurons that are immediately involved. It does not require the presence of long-lasting postsynaptic potentials, NMDA receptors, or other specialized details of biological neurons or synapses, although their inclusion in the model is likely to provide additional temporal stability [13]. Rather it points to complementary organizational mechanisms on the circuit level, which are likely to enhance the controllability and robustness of continuous attractors in neural circuits. The robustness of this learning-based model can be traced back to the fact that readout neurons can be trained to correct undesired circuit responses resulting from errors in their previous feedback. Furthermore, such error correction is not restricted to linear computational operations, since the previously demonstrated kernel property of these generic circuits allows even linear neurons to implement complex nonlinear control strategies through their feedback. As an example, we demonstrate in Figure 4 that even under biologically realistic high-noise conditions a linear readout can be trained to update a continuous attractor (Figure 4D), to filter out input activity during certain time intervals independent of the current state of the continuous attractor (Figure 4E), or to combine the time-varying analog variable encoded by the current state *CA*(*t*) of the continuous attractor with a time-varying variable *r*_1_(*t*) that is delivered by an online spike input. Hence, intention-based information processing [16] and other tasks that involve a merging of external inputs and internal state information can be implemented in this way. Figure 4C shows that a high-dimensional attractor need not entrain the firing activity of neurons in a drastic way, since it just restricts the high-dimensional–circuit dynamics **x**(*t*) to a slightly lower dimensional manifold of circuit states **x**(*t*) that satisfy **w** · **x**(*t*) = *f*(*t*) for the current target output *f*(*t*) of the corresponding linear readout. On the other hand, Figure 4E shows that the activity level *CA*(*t*) of the high-dimensional attractor can nevertheless be detected by other linear readouts, and can simultaneously be combined in a nonlinear manner with a time-varying variable *r*_2_(*t*) from one afferent circuit input stream, while remaining invariant to the other afferent input stream.

Finally, the same generic circuit also provides a model for the integration of evidence for decision making that is compatible with in vivo–like high noise conditions. Figure 4H depicts the timecourse of the same neural integrator as in Figure 4D, but here for the case where the rates *r*_1_,*r*_2_ of the two input streams assume in eight trials eight different constant values after the first 100 ms (while assuming a common value of 65 Hz during the first 100 ms). The resulting timecourse of the continuous attractor is qualitatively similar to the meandering path towards a decision threshold that has been recorded from neurons in area LIP where firing rates represent temporally integrated evidence concerning the dominating direction of random dot movements (see Figure 4A in [14]).

## Discussion

We have presented a theoretically founded model for real-time computations on complex input streams with persistent internal states in generic cortical microcircuits. This model does not require a handcrafted circuit structure or biologically unrealistic assumptions such as symmetric weight distributions, static synapses that do not exhibit pair-pulsed depression or facilitation, or neuron models with low levels of noise that are not consistent with data on in vivo conditions. Our model only requires the assumption that adaptive procedures (synaptic plasticity) in generic neural circuits can approximate linear regression. Furthermore, in contrast to classical learning paradigms for attractor neural networks, it is here not required that a large fraction of synaptic parameters in the circuit are changed when a new computational task is introduced or a new item is stored in working memory. Rather, it suffices if those neurons that provide the circuit output and a few neurons that provide feedback are subject to synaptic plasticity. Such minimal circuit modifications have the advantage that thereby created attractors of the circuit dynamics are high-dimensional. We have shown that the circuit state can simultaneously be in several of such high-dimensional attractors, and still retain sufficiently many degrees of freedom to absorb and process new information from online input streams. In particular, we have shown in Figures 2 and 4 how bottom-up processing can be reconfigured dependent on discrete internal states (implemented through high-dimensional attractors) by turning certain input channels on or off, and by changing the computational operations that are applied to input variables. Furthermore we have shown in Figure 4 that analog variables, which are extracted from an online input stream, can be combined in real-time computations with analog variables that are stored in high-dimensional continuous attractors. This provides in particular a model for the implementation of intention-based information processing [16] in cortical microcircuits.

It remains open how learning signals can induce neurons in a biological organism to compute specific linear feedback functions. But at least we have reduced this problem to the feasibility of perceptron-like learning (or more abstractly: to linear regression) for single neurons. Subsequent research will have to determine whether these learning requirements (which can be partially reduced to spike-timing dependent plasticity [46]) can be justified on the basis of results on unsupervised learning and reinforcement learning [47] in biological organisms.

Whereas it was previously already known that one can construct specific circuits that have universal computational capabilities for real-time computing on analog input streams, Theorems 1 and 2 of this article imply that a large variety of dynamical systems (in particular generic cortical microcircuits) can acquire through feedback such universal capabilities for computations that map time-varying inputs to time-varying outputs. It should be noted that these universal computational capabilities differ from the well-known but much weaker universal approximation property of feedforward neural networks (see [34]), since not only the static output of an arbitrary continuous static function is approximated, but also the dynamic response of arbitrary differential equations of higher-order to time-varying inputs.

The theoretical results of this article also provide an explanation for the astounding computational capability and flexibility of echo state networks [17]. In addition they can be used to analyze computational aspects of feedback in other biological dynamical systems besides neural circuits. Several such systems, for example, genetic regulatory networks, are known to implement complex maps from time-varying input streams (e.g., external signals) onto time-varying outputs (e.g., transcription rates). But little is known about the way in which these maps are implemented. Whereas feedback in biological dynamical systems is usually only analyzed and modeled from the perspective of control, we propose that an analysis of its computational aspects is likely to yield a better understanding of the computational capabilities of such systems.

## Materials and Methods

### Mathematical definitions, details to the proof of Theorem 1, and examples.

*Definition of feedback equivalence.* We recall that a *smooth* mapping is one for which derivatives of all orders exist (infinite differentiability), and that a *diffeomorphism* T: *T*: ℜ*^n^* → ℜ*^n^* is a smooth mapping for which there exists a well-defined smooth inverse *T* ^−1^: ℜ*^n^* → ℜ*^n^*.

Definition (see [33], Definition 5.3.1). Two *n*-dimensional systems **x**′ = *f*(**x**) + *g*(**x**)*v* and 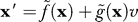 (with smooth vector fields 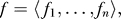 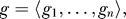 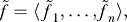 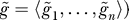 are called *feedback equivalent* (over the state space ℜ*^n^*) if there exists 1) a diffeomorphism *T*: ℜ*^n^* → ℜ*^n^*, and 2) smooth maps *α*,*β*: ℜ*^n^* → ℜ with *β*(**x**) ≠ 0 for all **x** ∈ ℜ*^n^*, such that, for each **x** ∈ ℜ*^n^*

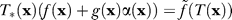
and

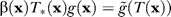
(where T_*_ denotes the Jacobian of *T*).

*Definition of the class S_n_.* Recall that a linear system **x′** = **Ax** + **b** *u* is *controllable* if it is possible to drive any state **x**_0_ to any other state **x**_1_ using an input (see [33], Definition 3.1.6). Controllability is a generic property of systems, and amounts to the requirement that the matrix (**b**,**Ab**,…,**A** *^n^* ^−1^ **b**) has full rank, where *n* is the dimension of the system (see [33], Theorem 2). Note that the linear system (Equation 7) satisfies this requirement, and hence is controllable.

We take *S_n_* to be the class of *n*-dimensional systems (Equation 3) that are *(globally) feedback linearizable*, that is to say, the systems (Equation 3) for which there exists some linear controllable system that is feedback equivalent to Equation 3 (see [33], Definition 5.3.2).

An *n*-dimensional system is feedback linearizable if and only if it is feedback-equivalent to the system (Equation 7) (see [33], Lemma 5.3.5). Therefore, we have the following:

Lemma: *A system (Equation 3), with smooth vector fields f = 〈f*_1_,…,*f_n_〉 and g =* 〈*g*_1_,…,*g_n_*〉 *belongs to S_n_ if and only if there exists a diffeomorphism T*: ℜ*^n^* → ℜ*^n^ and two smooth maps α*,*β*: ℜ*^n^* → ℜ*, with β*(**x**) ≠ 0 *for all* **x** ∈ ℜ*^n^, such that, for each* **x** ∈ ℜ*^n^*:

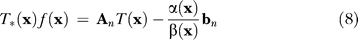

*and*

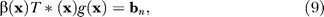

where T_*_ denotes the Jacobian of T and

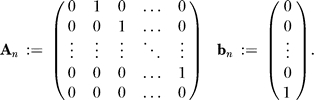



An interpretation of the property given in the above Lemma, that will be used in the proof of Theorem 1 in the section Details to the Proof of Theorem 1, is as follows (see [33], Chapter 5, for more discussion): For each input *μ*(*t*) and each solution *z*(*t*) of

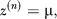
the vector function **x**(*t*) = *T* ^−1^(*Z*(*t*)) satisfies Equation 3 with the input *v*(*t*) = *α*(**x**(*t*)) + *β*(**x**(*t*))*μ*(*t*), where

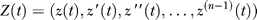



*Details to the proof of Theorem 1.* In this section, we prove the simulation result that is claimed in Theorem 1.

Take any system (Equation 3) in *S_n_* and any system (Equation 4) to be simulated. Using *T*,*α*,*β* as in the Lemma in section Definition of the Class *S_n_* that characterizes the class *S_n_*, we define:

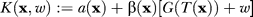
and we let *h*(**x**) be the first coordinate of *T*(**x**). In the special case where Equation 3 describes the dynamics of a circuit according to Equation 6, *α* is a linear function, *β* is a constant, and *T* is an invertible linear map from ℜ*^n^* to ℜ*^n^*.

Next, pick an external input *u*(*t*),*t* ≥ 0, and a solution *z*(*t*) of the forced system (Equation 4).

From the interpretation of feedback linearization given earlier (in the last part of Definition of the Class *S_n_*), it follows that for any inputs *u*(*t*) and *u*_0_(*t*) (in particular, one could take *u*_0_ ≡ 0), and each solution *z*(*t*) of

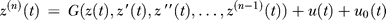
(that is, we use *μ*(*t*) = *G*(*z*(*t*),*z*′(*t*),*z*″(*t*),…,*z*(*n* ^−1^)(*t*)) + *u*(*t*) + *u*_0_(*t*) as the input to *z* ^(*n*)^ = *μ*), the vector function **x**(*t*) = *T* ^−1^(*Z*(*t*)) satisfies Equation 3 with input

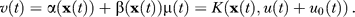
Furthermore, *Z*(*t*) = *T*(**x**(*t*)) means that *z*(*t*) = *h*(**x**(*t*)), as required for the notion of simulation.

This almost proves the simulation result, except for the fact that there is no reason for the initial value **x**(0) = *T* ^−1^(*Z*(0)) to be zero, since *z*(*t*) is an arbitrary trajectory. This is where the input *u*_0_ plays a role. Let *ξ* : = *T*(0). We will show that, given any solution *z*(*t*) and any input *u*(*t*), there is some input *u*_0_(*t*), with *u*_0_(*t*) ≡ 0 for all *t* ≥ 1, so that the solution of

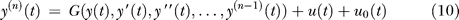
with *y*(0) = *ξ* has the property that *y*(*t*) = *z*(*t*) for all t ≥ 1. (Where *z*(*t*) is the desired trajectory to be simulated, with *u*_0_ ≡ 0.) Then letting **x**(*t*) = *T* ^−1^(*Y*(*t*)) instead of *T* ^−1^(*Z*(*t*)) means that **x**(0) and still *h*(**x**(*t*)) = *y*(*t*) = *z*(*t*) for all t ≥ 1.

Consider now an arbitrary solution *z*(*t*) of Equation 4 and let *ζ* be the vector with entries

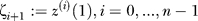
We next pick a scalar differentiable function *ϕ* such that *ϕ* ^(*i*)^(0) = *ξ_i_*_+1_ and *ϕ* ^(*i*)^(1) = *ξ_i_*_+1_ for *i* = 0,…,*n* − 1. (It is easy to see that such functions exist. For example, one may simply consider the linear system **p′** = **A** *_n_* **p** + **b** *_n_q* with states **p** and input q. This is a completely controllable linear system (cf. [33] Chapter 3), so we just pick an input *q*(*t*) that steers *ξ* into *ζ,* and finally let *ϕ*(*t*) be the first coordinate of **p**(*t*). Now we let

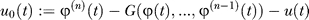
for *t* < 1, and *u*_0_(*t*) ≡ 0 for *t* ≥ 1, and claim that the solution of Equation 10 with *y*(0) = *ξ* has the property that *y*(*t*) = *z*(*t*) for all t ≥ 1. Since *u*(*t*) + *u*_0_(*t*) = *u*(*t*) for all t ≥ 1, we only need to show that *y* ^(*i*)^(1) = *z* ^(*i*)^(1) for every *i* = 0,…,*n* − 1. To see this, in turn, and using uniqueness of solutions of differential equations, it is enough to show that *y*(*t*): = *ϕ*(*t*) satisfies

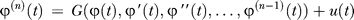
on the interval [0,1] and has derivatives at *t* = 0 as specified by the vector *ξ*. But this is indeed true by construction.

Finally, we remark that if | *u*(*t*) | ≤ *c* and | *z* ^(*i*)^ | (*t*) ≤ *c* for all *t* ≥ 0, then **x**(*t*) = *T* ^−1^(*Z*(*t*)) is bounded in norm by a constant that only depends on *c* (since *T* ^−1^ is continuous, by definition of diffeomorphism), and the numbers *b_i_*: = *z* ^(*i*)^(1) are also bounded by a constant that depends only on *c,* so *K*(**x**(*t*),*u*(*t*) + *u*_0_(*t*)) also is.

Corollary 3. *Analogous results can be shown for the simulation of systems consisting of any number k of higher order differential equations as in Equation 4. In this case fixed systems of first-order differential equations of a form as in Equation 3, but with k memoryless feedback functions K*_1_ *,…K_k_ that depend on the simulated higher-order system, can be shown to be able to simulate the dynamic response of arbitrary higher-order systems of differential equations.*

*Lie brackets.* The study of controllability and other properties of nonlinear systems is based upon the use of Lie bracket formalism and theory ([33], Chapter 4). We need this formalism to show in the section Application to Neural Network Equations that the class *S_n_* includes some neural networks of the form Equation 6. For any two vector fields *f* and g,

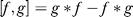
denotes the Lie bracket of *f* and *g*. Recall that the Lie bracket of two vector fields is a vector field that characterizes the effective direction of movement obtained by performing this “commutator” motion: follow the vector field *f* for *t* time steps, then *g* for *t* time steps, then *f* backward in time for *t* time steps, and finally *g* backward in time for *t* time steps, for small *t* > 0. To be more precise, denote formally by *e^tf^* the flow associated to *f*, and similarly for g. Consider the following curve, for any initial state *x*_0_:

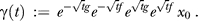
Applying repeatedly this expansion:

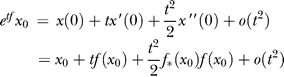
(and similarly for *g*), we obtain that

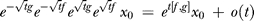
as *t* → 0, from which it follows that *γ*′(0) = [*f*,*g*](*x*_0_), which means that the direction of [*f*,*g*] is followed when performing the commutator motions. Using the possible noncommutativity of the vector fields, one generates in this manner genuinely new directions of movement in addition to those provided by the linear combinations of *f* and *g*. Well-known examples are provided by the Lie bracket of two rotations around orthogonal axes, which is a rotation around the remaining axis (see for example [33], page 150), or the motions involved in parking an automobile (see for example [33], Example 4.3.13).

Iterations of Lie brackets play a key role. Let us introduce, for any given vector field *f,* the operator *ad_f_,* which maps vector fields into vector fields by means of the formula ad*_f_*(*g*): = [*f*,*g*]. Iterations of the operator ad*_f_* are defined in the obvious way: 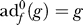 and 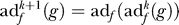 .

It is also useful to consider an operator *L_f_* that acts on scalar functions. We use the notation *L_f_ϕ,* for any (smooth) vector field *f* and (smooth) function *ϕ,* to denote the Lie derivative of *ϕ* along *f,* that is, ∇*ϕ* · *f*. The function *L_f_ϕ,* which is again smooth, is nothing more than the *directional derivative* of the function *ϕ* in the direction of the vector field *f,* in the sense of elementary calculus. One can also consider iterated applications of the operator *L_f_*.

*A characterization of S_n_ via lie brackets.* With these notations, we are ready to present a Lie geometric characterization of the class *S_n_*. The next theorem follows by combining the proofs of Proposition 5.3.9 and of Theorem 15 in [33] (with 0 = *X* = ℜ*^n^* in the notations of that text).

Theorem 4. The system **x′** = *f*(**x**) + *g*(**x**)*v is globally feedback linearizable if and only if there exists a smooth function*

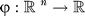
having everywhere nonzero gradient and satisfying the following properties: 1) *for each* **x** ∈ ℜ*^n^, the vectors* 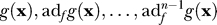 *are linearly independent;* 2) *for each* **x** ∈ ℜ*^n^ and each j =*0,…,*n −* 2, 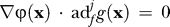 ; 3) *the map* 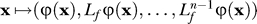 *is a bijection* ℜ*^n^ →* ℜ*^n^.*

Observe that the conditions amount to the existence of a well-behaved solution *ϕ* of a set of first-order linear partial differential equations. Existence of a solution of this form is not trivial to verify. To study solvability, in control theory one considers the following conditions:

**(LI)** The set of vector fields 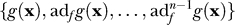 is linearly independent.
**(INV)** The distribution generated by 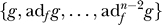 is involutive.

This last condition means that the Lie bracket of any two of the vector fields 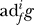 , for *i* ∈ {0,…,*n* − 2}, should be, for each **x**, a linear combination of these same *n* − 1 vectors.

One then has the following result (see Theorem 15 in [33]), which is a consequence of Frobenius' Theorem in partial differential equation theory: *A system satisfies both conditions* **(LI)** *and* **(INV)** *at a state* **x** *if and only if it is feedback linearizable in some open set containing* **x** *.* This provides a useful and complete characterization of local feedback linearizability, and in particular a necessary condition for global feedback linearizability. In examples, often these conditions lead one to a globally defined solution, see, e.g., example 5.3.10 in [33]).

*Application to neural network equations.* Let us now show with the help of Theorem 4 that the class *Sn* includes some fading-memory systems of the form Equation 6. Indeed, consider any system as follows:

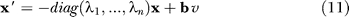
where the *λ_i_* ≠ *λ_j_* for each *i* ≠ *j* are all positive, diag(*λ*_1_,...,*λ*_n_) is the resulting diagonal matrix, and the column vector **b** = col(*b*_1_,…,*b*_n_) has nonzero entries: *b_i_* ≠ 0 for all *i*. (Such a system, which has the form Equation 6 with *ϕ*(*A* **x**) ≡ 0, consists of *n* first-order linear differential equations in parallel, and is obviously fading-memory.) It is easy to see that, up to signs (−1)*^i^*, we have

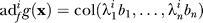
for *i* > 0, and the linear independence of 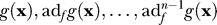 follows from the fact that these constant vectors form a Vandermonde matrix. Then we can pick *ϕ*(**x**) as a linear map **x** → **ax**, where **a** is any vector in ℜ*^n^* that is orthogonal to all of the vectors

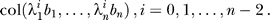
The map 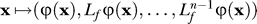 is represented then also by a Vandermonde matrix, so it is a bijection. Hence, conditions 1)−3) of Theorem 4 are satisfied, which implies that the system (Equation 11) belongs to the class *S_n_*.

As a further example, we now consider the following system, which also has the general form of the neural network Equation 6:

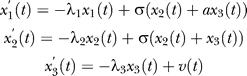
where *ϕ* is a scalar function, smooth but otherwise arbitrary for now, and *a* as well as the *λ_i_* are constants, also arbitrary for now. We will analyze this example using the Lie formalism described in the section Lie Brackets. The system has the form **x′** = *f*(**x**) + *g*(**x**)*v*, with *n* = 3, and *f* and *g* are the following vector fields:

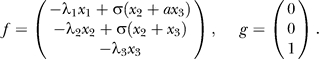
Note that the Jacobian g_*_ of *g* is identically zero, which simplifies the computation of Lie brackets. We calculate ad*_f_g*(**x**) = [*f*,*g*](**x**) = −f_*_(**x**)*g*(**x**) and [*g*,ad*_f_g*] = (ad*_f_g*)_*_(**x**)g(**x**) as follows:

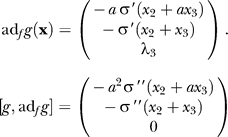
The involutivity condition says that the set of vector fields {*g*,ad*_f_g*} should be involutive, which means that [*g*,ad*_f_g*](**x**) should be in the span of *g*(**x**) and ad*_f_g*(**x**) for all **x**. Let us evaluate *g*,ad*_f_g*,[*g*,ad*_f_g*] at the particular points for which *x*_3_ = 0, so that we obtain, respectively, three vectors *v*_1_,*v*_2_,*v*_3_ that depend on *x*_2_ only:

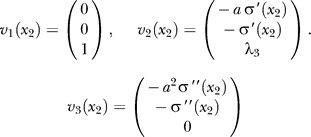
If [*g*,ad*_f_g*](**x**) is in the span of *g*(**x**) and ad*_f_g*(**x**) for all vectors **x**, then, in particular, *v*_3_(*x*_2_) must belong to the span of *v*_1_(*x*_2_) and *v*_2_(*x*_2_) for all *x*_2_. This means that there is, for each *x*_2_, a scalar *r*(*x*_2_) such that

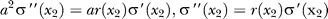
If *a* ≠ 0, it follows that *ϕ*″(*x*_2_) = *aϕ*″(*x*_2_) for all *x*_2_. So, if also *a* ≠ 1, we conclude that *ϕ*″(*x*_2_) must vanish for all *x*_2_. Thus, the system in our example (assuming *a* ∉ {0,1}) is feedback linearizable only if *ϕ* is a linear function.

On the other hand, consider now the cases *a* = 0 or *a* = 1. Then, the involutivity condition becomes the requirement that there should exist a scalar function *r* such that

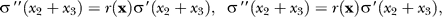
which can be achieved provided only that the function *ϕ*′ is everywhere nonzero (which is true if *ϕ* is, for example, a standard sigmoidal function), simply by taking *r*(**x**) = *ϕ*″(*x*_2_ + *x*_3_) / *ϕ*′(*x*_2_ + *x*_3_). The linear independence condition amounts to showing that the set of vectors {*g*,ad*_f_g*,ad^2^ *_f_g*} is linearly independent. Computing the determinant of the matrix that has these vectors as columns, when *a* = 0 we obtain −*ϕ*′(*x*_2_)[*ϕ*′(*x*_2_ + *x*_3_)]^2^, which is everywhere nonzero, provided that we again assume that *ϕ* has an everywhere nonzero derivative. Thus, the Lie-theoretic conditions for feedback linearization are satisfied, for any choice of *λ_i_*, when *a* = 0. In the case *a* = 1, the same computation gives a determinant of *λ*_1_ − *λ*_2_)[*ϕ*′(*x*_2_ + *x*_3_)]^2^, so the Lie-theoretic conditions for feedback linearization are satisfied, for any choice of *λ_i_* such that *λ*_1_ ≠ *λ*_2_.

#### Mathematical definitions and details to the proof of Theorem 2.

*Fading-memory filters.* A map (or filter) *F* from input to output streams is defined to have *fading memory* if its current output at time *t* depends (up to some precision *ɛ*) only on values of the input **u** during some finite time interval [*t* – *T*,*t*]. (We use in this section boldface letters to denote input streams, because they typically have a dimension larger than 1.) In formulas: *F* has fading memory if there exists for every *ɛ* > 0 some *δ* > 0 and *T* > 0 so that | (*F* **u**)(*t*) − (*F* **ũ**)(*t*) | < *ɛ* for any t ∈ ℜ and any input functions **u**,**ũ** with || **u**(*τ*) − **ũ**(*τ*) || < *δ* for all *τ* ∈[*t* − *T*,*t*]. This is a characteristic property of all filters that can be approximated by an integral over the input stream **u**, or more generally by Volterra or Wiener series. Note that nontrivial Turing machines and FSMs *cannot* be approximated by filters with fading memory, since they require a persistent memory.

*Finite state machines.* The deterministic *finite state machine* (FSM), also referred to as deterministic finite automaton, is a standard model for a digital computer, or more generally for any realistic computational device that operates in discrete time with a discrete set of inputs and internal states [26]. One assumes that an FSM is at any time in one of some finite number *l* of states, and that it receives at any (discrete) time step one input symbol from some alphabet {*s*_1_,…,*s_k_*} that may consist of any finite number *k* of symbols. Its “program” may consist of any transition function *TR*:{*s*_1_,…,*s_k_*} × {1,…,*l*} → {1,…,*l*}, where *TR*(*s*_i_,*j*′) = *j* denotes the new internal state *j* which the FSM assumes at the next time step after processing input symbol *s_i_* in state j′.

*Precise statement of Theorem 2.* We consider here a slight variation of the FSM model, which is more adequate for systems that operate in continuous time and receive analog inputs (for example, trains of spikes in continuous time). We assume that the raw input is some arbitrary *n*-dimensional input stream **u** (i.e., **u**(*t*) ∈ ℜ*^n^* for every *t* ∈ ℜ*^n^*). Furthermore we assume that there exist pattern detectors *F*_1_,…,*F_k_* that report the occurrence of spatio–temporal patterns in the input stream **u** from *k* different classes *C_1_*,…,*C*_k_. In the case where the input **u** consists of spike trains, these classes could consist, for example, of particular patterns of firing rates, of particular spike patterns, or of particular correlation patterns among some of the input spike trains. It was shown in [5] that readouts from generic neural microcircuit models can easily be trained to approximate the role of such pattern detectors *F*_1_,…,*F_k_*. We assume that the detection of a pattern from class *C_i_* by pattern detector *F_i_* affects the state of the FSM according to its transition function *TR* in a way that corresponds to the presentation of input symbol *s_i_* in the discrete-time version: if *j*′ was its preceding state, then it changes now within some finite switching time to state j = *TR*(*s_i_*,*j*′).

To make an implementation of such FSM by a noisy system feasible, we assume that the pattern detectors (*F*_1_ **u**)(*t*),…,(*F_k_* **u**)(*t*) always assume values ≤0, except during a switching episode. During a switching episode, exactly one of the pattern detectors (*F_i_* **u**)(*t*) assumes values >0. We assume that this (*F_i_* **u**(*t*) reaches values ≥1 during this switching episode. We also assume that the length of each switching episode (i.e., the time during which some (*F_i_* **u**(*t*) assumes values >0) is bounded from above by some constant *δ,* and that the temporal distance between the beginnings of any two different switching episodes is at least Δ + 3δ′ (where Δ is the assumed temporal delay of the feedback in the circuit). To avoid that the subsequent construction is based on unrealistic assumptions, we allow that each pattern detector *F_i_* is replaced by some arbitrary filter 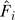 so that 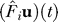 is a continuous function of time (with values in some arbitrary bounded range [−*B*,*B*]) with 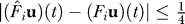 for any input stream **u** that is considered.

The informal statement of Theorem 2 is made precise by the subsequent Theorem 5 (see Figure 6 for an illustration). It exhibits a simple construction method whereby fading-memory filters with additive noise of bounded amplitude can be composed into a closed loop system *C* that emulates an arbitrary given FSM in a noise-robust manner. The resulting system *C* can be embedded into any other fading-memory system, which receives the outputs *CL* – *Ĥ_j_*(*t*) of *C* as additional inputs. In this way, any given fading-memory system can integrate the computational capability and nonfading states of the FSM that is emulated by *C* into its own real-time computation on time-varying input streams **u**.

**Figure 6:**
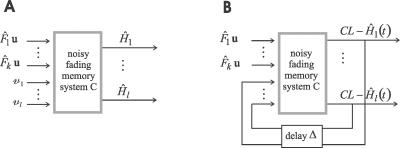
Emulation of an FSM by a Noisy Fading-Memory System with Feedback According to Theorem 5 (A) Underlying open-loop system with noisy pattern detectors 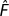
_1_, …, 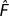 *_k_* and suitable fading-memory readouts *Ĥ*_1_, …, *Ĥ_l_* (which may also be subject to noise). (B) Resulting noise-robust emulation of an arbitrary given FSM by adding feedback to the system in (A). The same readouts as in (A) (denoted *CL* − *Ĥ_j_*(*t*) in the closed loop) now encode the current state of the simulated FSM.

An essential aspect of the proof of Theorem 5 is that suitable fading-memory filters *H_j_* can prevent in the closed loop the accumulation of errors through feedback, even if the ideal fading-memory filters *H_j_* are subsequently replaced by imperfect approximations *Ĥ_j_*. One just has to construct the ideal fading-memory filters *H_j_* in such a way that they take into account that their previous outputs, which have been fed back into the system *C,* may have been corrupted by additive noise. As long as this additive noise of bounded amplitude has not been amplified in the closed loop, the filters *H_j_* can still recover which of the finitely many states of the emulated FSM *A* was represented by that noise-corrupted feedback.

From the perspective of neural circuit models, it is of interest to note that the construction of the system *C* can be replaced by an adaptive procedure, whereby readouts from generic cortical microcircuit models are trained to approximate the target filters *H_j_*. General approximation results [4,5,37] imply that if the neural circuit is sufficiently large and contains sufficiently diverse components (for example, dynamic synapses with slightly different parameter values), then the actual outputs *Ĥ_j_* of these readouts can approximate the target filters *H_j_* uniformly up to any given maximal error *ɛ* > 0. Theorem 5 guarantees that the resulting neural circuit model with these (imperfectly) trained readouts can in the closed loop emulate the given FSM *A* in a reliable manner, provided that the neural circuit model is sufficiently large and diverse so that its readout can achieve an approximation error *ɛ* not larger than 1/4.

Theorem 5. *One can construct for any given FSM A, some time-invariant fading-memory filters H*_1_ *,…,H_l_ with the property that any approximating filters Ĥ*_1_,…,*Ĥ_l_ with |H_j_ − Ĥ_j_* | ≤ 1/4 *provide in the closed loop with delay* Δ *(see Figure 6) outputs CL − Ĥ_1_*,…,*CL − Ĥ_l_ that simulate the FSM A in the following sense:*

*If* [*t*_1_,*t*_2_] *is some arbitrary time interval between switching episodes of the FSM A with noise-free pattern detectors* (*F*_1_ **u**)(*t*),…,(*F_k_* **u**)(*t*) *during which A is in state j, then the outputs CL − Ĥ_i_(t) of the approximating filters Ĥ_i_ in the closed loop with noisy pattern detectors* 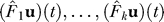 *satisfy CL − Ĥ_j_*(*t*) ≥ 3/4 and CL − Ĥ_j*_(*t*) ≤ 1/4 for all j^*^ ≠ j and all t ∈ [*t*_1_,*t*_2_]*.*

*Proof of the precise statement of Theorem 2.* We present here a proof of Theorem 5 (see Precise Statement of Theorem 2 section above), which provides a formally precise version of Theorem 2.

To prove that the given FSM *A* can be implemented in a noise-robust fashion, we construct suitable time-invariant fading-memory filters *H*_1_,…,*H_l_*. They receive as inputs the time-varying functions 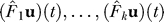 . In addition, they receive in the open-loop inputs *v*_1_(*t*),…,*v_1_*(*t*), where each *v_j_*(*t*) will be replaced by a delayed version of the output of *H_j_* (or *Ĥ_j_*) in the closed loop (see Figure 6). The filters *H_j_* will be defined in such a way that *H_j_*(*t*) ≥ 1 signals in the closed loop that the FSM *A* is at time *t* in state *j*. To make this implementation noise-robust, we make sure that even if one replaces the filters *H_j_* by noisy approximations *Ĥ_j_*, which satisfy in the open loop | *H_j_*(*t*) − *Ĥ_j_*(*t*) | ≤ ¼ (for all *t* ∈ ℜ and any time-varying inputs 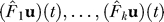 and *v*_1_(*t*),…,*v_l_*(*t*)), then the closed-loop version of such imperfect approximations *Ĥ_j_* simulates the FSM *A* in such a way that *Ĥ_j_*(*t*) ≥ ⅓ implies that *A* is in state *j* at time *t*.

Let Δ be the time delay in the feedback for the closed loop. We now define the target outputs *H*_1_(*t*),…,*H_l_*(*t*) (for the open-loop version, where the *H_j_* receive in addition to 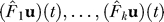 some arbitrary time-varying variables *v*_1_(*t*),…,*v_1_*(*t*) with values in [−1,2] as inputs). We define the target outputs of *H*_1_,…,*H_l_* as a stationary transformation of the time-varying inputs *v_j_*(*t*) and of the outputs of the following two other types of time invariant fading-memory filters: (i) 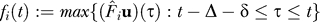 for *i* = 1,…,*k;* (ii) *v_j_*(*t −* 2*δ) for j =* 1*,…,l*. We will show below in Lemma 6 and Lemma 7 that both of these functions of time can be viewed as outputs of time-invariant fading-memory filters that receive as inputs the time-varying functions 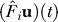 (for some arbitrary input stream *u*) and *v_j_*(*t*). On the basis of these two Lemmata, it is clear that the *H_j_* are time-invariant fading-memory filters if one can define *H*_1_(*t*),…,*H_l_*(*t*) as (static) continuous functions of the variables *v_j_*(*t*) and the outputs of the filters (i) and (ii). In the following we sometimes refer to *H*_1_,…,*H_l_* as static functions of input vectors (*f*_1_(*t*),…,*f_k_*(*t*)*v*_1_(*t*),…,*v_l_*(*t*),*v*_1_(*t* − 2*δ*),…,*v_l_*(*t* − 2*δ*)) from ℜ*^k^* ^+2*l*^, and sometimes as filters with time-varying inputs 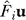 and *v_j_* (if we view the filters (i) and (ii) as being part of the computation of *H_j_*). To define such functions *H_j_*(*t*), we first define for each *j* ∈ {1,…,*l*} two disjoint closed and bounded sets *S_j_*_,0_,*S_j_*_,1_ ∈ ℜ*^k^* ^+2*l*^, and we set *H_j_*(**x**) = 0 for **x** ∈ *S_j_*_,0_ and *H_j_*(**x**) = 1 for **x** ∈ *S_j_*_,1_. Since the sets *S_j_*_,0_ and *S_j_*_,1_ will have positive distance (i.e., inf{|| **x** − **y** ||**:** **x** ∈*S_j_*_,0_ and **y** ∈ *S_j_*_,1_} > 0), it follows from standard arguments of analysis that the definition of *H_j_* can be continued outside of *S_j_*_,0_,*S_j_*_,1_ to yield a continuous function from ℜ*^k^* ^+2*l*^ into ℜ.

To define the sets *S_j_*_,0_,*S_j_*_,1_, we consider the following two types of conditions:

(*A_j_*) There exist *i* ∈{1,…,*k*} and *j*′ ∈{1,…,*l*} so that *TR*(*i*,*j*′) = *j, f_i_*(*t*) ≥ ⅓ and *f_i_*_′_(*t*) ≤ ¼ for all *i*′ ≠ *i*, *v_j′_*(*t* − 2*δ*) ≥ ⅓ and v_j__*_(*t* − 2*δ*) ≤ ¼ for all j^*^ ≠ *j*′.

(*B_j_*)*f_i_*(*t*) ≤ ¼ for *i* = 1,…,*k*, *v_j_*(*t*) ≥ ⅓ and v_j__*_(*t*) ≤ ¼ for all j^*^ ≠ *j*.

We say that a vector (*f*_1_(*t*),…,*f_k_*(*t*),*v*_1_(*t*),…,*v_l_*(*t*),*v*_1_(*t* − 2*δ*),…,*v_l_*(*t* − 2*δ*)) ∈[−*B*,*B*]*^k^* × [−1,2]^2*l*^ belongs to set *S_j_*_,1_ if the conditions *A_j_* or *B_j_* apply, and to set *S_j_*_,0_ if there exists some j^*^ ≠ *j* so that the conditions A_j__*_ or B_j__*_ apply.

It follows immediately from the definition of the sets *S_j_*_,0_ and *S_j_*_,1_ that they are closed and bounded. One can also verify immediately that for any *j*,*j*′ ∈ {1,…,*l*} the ∈ conditions *A_j_* and *B_j_*_′_ can never be simultaneously satisfied (for any values of the variables *f_i_*(*t*),*v_j_*(*t*),*v_j_*(*t* − 2*δ*)). In addition the conditions *A_j_* and *A_j_*_′_ (*B_j_* and *B_j_*_′_) can never be simultaneously satisfied for any *j* ≠ *j*′. This implies that the sets *S_j_*_,0_ and *S_j_*_,1_ are disjoint for each *j* ∈ {1,…,*l*}.

We define for each *j* ∈ {1,…,*l*} a continuous function *H_j_*: ℜ*^k^* ^+2*l*^ → [0,1] by setting

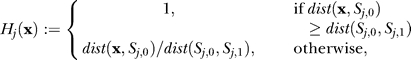
where *dist*(**x**,*S*): = inf{|| **x** − **y** ||: *y* ∈ *S*} for any set *S* ∈ ℜ*^k^* ^+2*l*^. It is then obvious that *H_j_* is a continuous function from ℜ*^k^* ^+2*l*^ into [0,1] with *H_j_*(**x**) = 0 for all **x** ∈ *S_j_*_,0_ and *Hj*(**x**) = 1 for all **x** ∈ *S_j_*_,1_. These functions *H_j_* will prevent the amplification of noise in the closed loop, since they assume outputs 1 or 0 in all relevant situations, even if their inputs deviate by up to ¼ from their “ideal” values.

We consider some arbitrary imprecise and/or noisy versions *Ĥj* of these filters *Ĥj* (with inputs 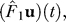 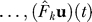 and additional inputs *v*_1_(*t*),…,*v_l_*(*t*) whose output differs at any time *t* by at most ¼ from that of *H_j_* (of course in the closed loop these deviations could be accumulated and amplified to values >¼). We want to show that for any such *Ĥ*_1_,…,*Ĥ_l_* the closed loop version of the circuit implements the given FSM *A*. As initial condition we assume that the given FSM *A* is in state 1 for *t* ≤ 0, and consequently also that *Ĥ*_1_(*t*) ≥ ⅓ and *Ĥ_j_*(*t)* ≤ ¼ for *j* = 2,…,l, as well as *f_i_*(*t*) ≤ ¼ for all *t* ≤ 0 and *i* = 1,…,*k*.

We will now prove the claim of Theorem 5 for arbitrary time intervals [*t*_1_,*t*_2_] outside of switching episodes. We assume without loss of generality that *t*_2_ marks the beginning of the next switching episode [*t*_2_,*t*_3_] for some *t*_3_ > *t*_2_ with | *t*_3_ − *t*_2_ | ≤ *δ.* Furthermore we assume that either *t*_1_ = 0 (Case 1), or *t*_1_ is the endpoint of the preceding switching episode [*t*_0_,*t*_1_] with | *t*_1_ − *t*_0_ | ≤ δ (Case 2). The formal proof is carried out by induction on the number of preceding switching episodes (and Case 2 represents the induction step). In both cases one just needs to analyze the outputs of the previously defined filters 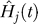 in the case where some of their inputs are delayed feedbacks of their previous outputs.

Case 1: *t*_1_ = 0. We prove by a nested induction on *m* ∈
N that *CL* − *Ĥ*_1_(*t*) ≥ ⅓ and CL − *Ĥ_j_*(*t*) ≤ ¼ for all *j* > 1 holds for all *t* ∈ [*m* · Δ,(*m* + 1) · Δ) ∩[*t*_1_,*t*_2_]. Since by assumption no switching episode occurs during [*t*_1_,*t*_2_], one has *f_i_*(*t*) ≤ ¼ for *i* = 1,…,*k* and for all *t* ∈ [*t*_1_,*t*_2_]. Furthermore, by our assumption on the initial condition of the FSM *A* (for *m* = 0), or by the induction hypothesis of the nested induction (for *m* > 0), we can assume that the variables *v_j_*(*t*) of the open loop have now been assigned in the closed loop the values CL – *Ĥ_j_*(*t* − Δ); therefore, they are ≥⅓ for *j* = 1 and ≤¼ for all *j* > 1. Hence condition *B*_1_ in the definition of the sets *S_j_*,_0_,*S_j_*_,1_ applies, and the current circuit input is therefore in *S*_1,1_. Thus *H*_1_ = 1 and *H_j_* = 0 for *j* > 1, which implies *Ĥ*_1_ ≥ ⅓ and *Ĥ_j_* ≤ ¼ for *j* > 1 in the open loop, hence *CL* − *Ĥ*_1_(*t*) ≥ ⅓ and *CL* − *Ĥj*(*t*) ≤ ¼ for *j* > 1 in the closed loop (since *v_j_*(*t*) = *CL* − *Ĥ_j_*(*t* − Δ) in the closed loop).

Case 2: *t*_1_ is the endpoint of a preceding switching episode [*t*0,*t*_1_]. Assume that 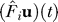 is the (approximating) pattern detector that assumes a value ≥¾ during the preceding switching episode [*t*_0_,*t*_1_], while 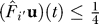 for all *i*′ ≠ *i* during [*t*_0_,*t*_1_]. Let *t*′ ∈[*t*_0_,*t*_1_] be the first timepoint where 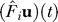 reaches a value ≥¾. Then *f_i_*(*t*) ≥ ¾ and f_i*_(*t*) ≤ ¼ for all i^*^ ≠ *i* and for all t ∈ [*t*′,*t*′ + Δ + *δ*] (by the definition of the filters *f_i_*(*t*)). Furthermore, one has by the induction hypothesis that for the state *j*′ in which the FSM *A* was before the switching episode [*t*_0_,*t*_1_] that *CL* − *Ĥ_j_*_′_(*t* − Δ − 2*δ* ≥ ¾ and *CL* − Ĥ_j*_(*t* − Δ − 2*δ*) ≤ ¼ for all *j* ^*^ ≠ *j*′ and all t ∈ [*t*′,*t*′ + Δ + 2*δ*]. We exploit here that *t*_0_ ≤ *t*′ ≤ *t*_1_ ≤ *t*_0_ + *δ*, hence *t*_0_ − Δ − 2*δ* ≤ *t* − Δ − 2*δ* ≤ *t*_0_ for all t ∈ [*t*′,*t*′ + Δ + *δ*]. Furthermore, we have assumed that the minimal distance between the beginnings of switching episodes is Δ + 3*δ*. Therefore, the considered range [*t*_0_ − Δ − 2*δ*,*t*_0_] for *t* − Δ − 2*δ* is contained in the preceding time interval before the switching episode [*t*_0_,*t*_1_] to which the induction hypothesis applies.

The previously listed conclusions imply that for *t* ∈ [*t*′,*t*′ + Δ + *δ*] the current input to the open loop lies in the set *S_j_*_,1_ for *j* = *TR*(*i*,*j*′), hence *H_j_* = 1 and *Ĥ_j_* ≥ ¾, while H_j*_ = 0 and Ĥ_j*_ ≤ ¼ for all other j^*^. But if one chooses as inputs *v*_1_(*t*),…,*v_l_*(*t*) to the open loop just those values which the circuit receives in the closed loop, one gets *CL* − *Ĥ_j_*(*t*) ≥ ¾ and *CL* − Ĥ_j*_(*t*) ≤ ¼ for all j^*^ ≠ *j* and all *t* ∈ [*t*′,*t*′ + Δ + *δ*], in particular for all *t* ∈[*t*_1_,*t*_1_ + Δ].

One can then prove by a nested induction on *m* ∈
N like in Case 1 that the outputs *CL* − Ĥ_j*_(*t*) for j^*^ = 1,…,*l* have the desired values for *t* ∈[*t*_1_ + *m*Δ,*t*_1_ + (*m* + 1) · Δ]∩ [*t*_1_,*t*_2_]. The preceding argument provides the verification of the claim for the initial step *m* = 0 of this nested induction.

To complete the proof of Theorem 5, it only remains to verify the following two simple facts about time-invariant fading-memory filters.

Lemma 6. Assume that 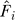 *is some arbitrary time-invariant fading-memory filter, and Δ*,*δ are arbitrary positive constants. Then the map that assigns to an input stream* **u** *the function* 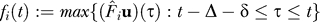 *is also a time-invariant fading-memory filter.*

Proof of Lemma 6: Assume some *ɛ* > 0 is given. Fix *δ*′ and *T* > 0 so that 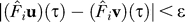 for all *τ* ∈ [*t* − Δ − *δ*,*t*] and all **u,v** with || **u**(*s*) − **v**(*s*) || < **δ′** for all *s* ∈ [*t* − Δ − *δ* − *T*,*t*].

Then |max{( *F̂* *_i_* **u**)(τ): *t* − Δ − δ ≤ τ ≤ *t*} −max{( *F̂* *_i_* **v**)(τ): *t* − Δ − δ ≤ τ ≤ *t*}| < ɛ.

Lemma 7. *The filter that maps for some arbitrary fixed δ* > 0 *the function u*(*t*) *onto the function u*(*t −* 2*δ*) *is time-invariant and has fading memory.*

Proof of Lemma 7 follows immediately from the definitions (choose T ≥ 2*δ* in the condition for fading memory).

This completes the proof of Theorem 5, which shows that any given FSM can be reliably implemented by fading-memory filters with feedback even in the presence of noise.

Remark. In the application of this theory to cortical microcircuit models, we train readouts from such circuits to *simultaneously* assume the role of the pattern detectors 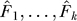 , which become active if some pattern occurs in the input stream that may trigger a state change of the simulated FSM *A*, *and* the role of the fading-memory filters *Ĥ*_1_,…,*Ĥ_l_*, which create high-dimensional attractors of the circuit dynamics that represent the current state of the FSM *A*.

#### Details of the cortical microcircuit models.

We complement in this section the general description of the simulated cortical microcircuit models from the section Applications to Generic Cortical Microcircuit Models, providing in particular all missing data that are needed to reproduce our simulation results. The original code that was used for these simulations is online available at http://www.lsm.tugraz.at/research/index.html.

Each circuit consisted of 600 neurons, which were placed on the integer grid points of a 5 × 5 × 24 grid. Twenty percent of these neurons were randomly chosen to be inhibitory. The probability of a synaptic connection from neuron *a* to neuron *b* (as well as that of a synaptic connection from neuron *b* to neuron *a*) was defined as *C* · exp(−*D* ^2^(*a*,*b*)/*λ* ^2^), where *D*(*a*,*b*) is the Euclidean distance between neurons *a* and *b*, and *λ* is a parameter that controls both the average number of connections and the average distance between neurons that are synaptically connected (we set *λ* = 3.). Depending on whether the pre- or postsynaptic neuron was excitatory (*E*) or inhibitory *(I),* the value of *C* was set according to [44] to 0.3 *(EE),* 0.2 *(EI),* 0.4 *(IE),* 0.1 *(II),* yielding an average of 10,900 synapses for the chosen circuit size. External inputs and feedbacks from readouts were connected to populations of neurons in the circuit with randomly chosen connection strengths.

I&F neurons. A standard leaky I&F neuron model was used, where the membrane potential *V_m_* of a neuron is given by:

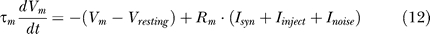
where *t_m_* is the membrane time constant (30 ms), which subsumes the time constants of synaptic receptors as well as the time constant of the neuron membrane. Other parameters are: absolute refractory period 3 ms (excitatory neurons), 2 ms (inhibitory neurons); threshold 15 mV (for a resting membrane potential V*_resting_*, assumed to be 0), reset voltage drawn uniformly from the interval [13.8 mV, 14.5 mV] for each neuron; input resistance *R_m_*, 1 MΩ, constant nonspecific background current *I_inject_* uniformly drawn from the interval [13.8 mV, 14.5 mV] for each neuron; input resistance R*_m_*, 1 MΩ, constant nonspecific background current I*_inject_* uniformly drawn from the interval [13.5 nA, 14.5 nA] for each neuron; additional time-varying noise input current *I_noise_* drawn every 5 ms from a Gaussian distribution with mean 0; and SD chosen randomly for each neuron from the uniform distribution over the interval [4.0 nA, 5.0 nA]. For each simulation, the initial condition of each I&F neuron, i.e., its membrane voltage at time *t* = 0, was drawn randomly (uniform distribution) from the interval [13.5 mV, 14.9 mV]. Finally, I*_syn_*(*t*) is the sum of input currents supplied by the explicitly modeled synapses.

HH neurons: We used single-compartment HH neuron models with passive and active properties modeled according to [48,49]. The membrane potential was modeled by

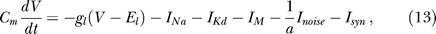
where *C_m_* = 1*μF*/*cm* ^2^ is the specific membrane capacitance, *g_L_* = 0.045 *mS*/*cm* ^2^ is the leak conductance density, *E_L_* = −80 *mV* is the leak reversal potential, and *I_syn_*(*t*) is the input current supplied by explicitly modeled synapses (see the definition below). The membrane area *a* of the neuron was set to be 34,636 *μm* ^2^ as in [48]. The term *I_noise_*(*t*) (see the precise definition below) models smaller background input currents from a large number of more distal neurons, causing a depolarization of the membrane potential and a lower input resistance commonly referred to as “high conductance state” (for a review see [42]).

In accordance with experimental data on neocortical and hippocampal pyramidal neurons ([50–53]) the active currents in the HH neuron model comprise a voltage dependent *Na* ^+^ current *I_Na_* ([54]) and a delayed rectifier *K* ^+^ current *I_Kd_* ([54]). For excitatory neurons, a noninactivating *K* ^+^ current *I_M_* ([55]) responsible for spike frequency adaption was included in the model.

The voltage-dependent *Na* ^+^ current was modeled by:

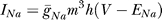


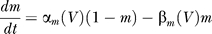


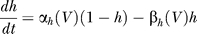


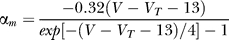


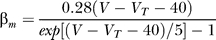


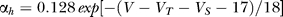


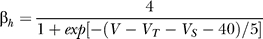
where *V_T_* = −63 *mV*, and the inactivation was shifted by 10 *mV* toward hyperpolarized values (*V_S_* = 10 *mV*) to reflect the voltage dependence of *Na* ^+^ currents in neocortical pyramidal cells [56]. The peak conductance densities for the *I_Na_* current was chosen to be 500 *pS*/*μm* ^2^.

The delayed rectifier *K* ^+^ current was modeled by:

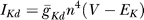


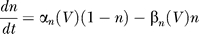


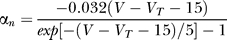


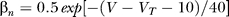
The peak conductance densities for the *I_Kd_* current was chosen to be 100 *pS*/*μm* ^2^.

The noninactivating *K* ^+^ current was modeled by:

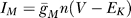


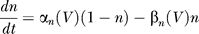


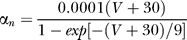


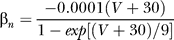
The peak conductance density for the *I_M_* current was chosen to be 5 *pS*/*μm* ^2^.

For each simulation, the initial condition of each neuron, i.e., the membrane voltage at time *t* = 0, was drawn randomly (uniform distribution) from the interval [−70, −60] mV.

The total **synaptic background current**, *I_noise_*(*t*), was a sum of two independent currents:

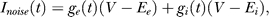
where *g_e_*(*t*) and *g_i_*(*t*) are time-dependent excitatory and inhibitory conductances. The values of respective reversal potentials were *E_e_* = 0 *mV* and *E_i_* = −75 *mV* mV.

The conductances *g_e_*(*t*) and *g_i_*(*t*) were modeled according to [48] as a one-variable stochastic process similar to an Ornstein−Uhlenbeck process:

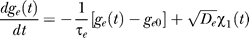


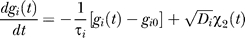
where *g_e_*_0_ = 0.012 *μS* and *g_i_*_0_ = 0.057 *μS* are average conductances, *τ_e_* = 2.7 ms and *τ_e_* = 10.5 ms are time constants, *D_e_* = 0.0067 *μS* ^2^/s and *D_i_* = 0.0083 *μS* ^2^/s are noise-diffusion constants, *χ*_1_(*t*) and *χ*_2_(*t*) are Gaussian white noise of zero mean and unit standard deviation.

Since these stochastic processes are Gaussian, they can be integrated by an exact update rule:

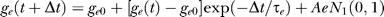


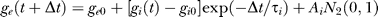
where *N*_1_(0,1) and *N*_2_(0,1) are normal random numbers (zero mean, unit SD) and *A_e_* and *A_i_* are amplitude coefficients, given by:

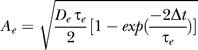


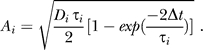



According to [48], this model captures the spectral and amplitude characteristics of the input conductances of a detailed biophysical model of a neocortical pyramidal cell that was matched to intracellular recordings in cat parietal cortex in vivo. Furthermore, the ratio of the average contributions of excitatory and inhibitory background conductances was chosen to be five in accordance with experimental studies during sensory responses [57–59]. The maximum conductances of the synapses were chosen from a Gaussian distribution with a SD of 70% of its mean (with negative values replaced by values chosen from an uniform distribution between 0 and two times the mean).

We modeled the (short-term) **dynamics of synapses** according to the model proposed in [43], with the synaptic parameters *U* (use), *D* (time constant for depression), and *F* (time constant for facilitation) randomly chosen from Gaussian distributions that model empirically found data for such connections (see in Methods, Details of the Cortical Microcircuit Models). This model predicts the amplitude *A_k_* of the EPSC for the *k^th^* spike in a spike train with interspike intervals Δ_1_,Δ_2_,Δ*_k_*_−1_ through the equations

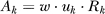


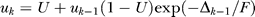


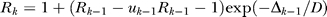
with hidden dynamic variables *u* ∈ [0,1] and *R* ∈ [0,1], whose initial values for the first spike are *u*_1_ = *U* and *R*_1_ = 1 (see [60] for a justification of this version of the equations, which corrects a small error in [43]).

The postsynaptic current for the *k^th^* spike in a presynaptic spike train that had been generated at time *t_k_*, is modeled for *t* ≥ *t_k_* + Δ (where Δ is the transmission delay) by *A_k_* exp(−(*t* − *t_k_* − Δ)/*τ_s_*) with *τ_s_* = 3 ms (*τ_s_* = 6 ms) for excitatory (inhibitory) synapses. The transmission delays Δ between neurons were chosen uniformly to be 1.5 ms for EE-connections, and 0.8 ms for the other connections. The total **synaptic input current** *i_syn_*(*t*) was modeled by the sum of these currents for all synapses onto a neuron.

Synaptic parameters. Depending on whether *a* and *b* were excitatory *(E)* or inhibitory *(I),* the mean values of the three parameters *U*,*D*,*F* (with *D*,*F* expressed in seconds, s) were chosen according to [44] to be .5, 1.1, .05 *(EE),* .05, .125, 1.2 *(EI),* .25, .7, .02 *(IE),* .32, .144, .06 (*II*). The SD of each of these parameters was chosen to be 50% of its mean. The mean of the scaling parameter *w* (in nA) was chosen to be 70 (EE), 150 (EI), −47 (IE), −47 (II). The SD of the parameter *w* was chosen to be 70% of its mean and was drawn from a gamma distribution. In the case of input synapses, the parameter *w* had a value of 70 nA if projecting onto a excitatory neuron and −47 nA if projecting onto an inhibitory neuron.

The synaptic weights **w** of **readout neurons** were computed by linear regression to minimize the mean squared error (**w** · **x**(*t*) – *f*(**t**))^2^ with regard to a specific target output function *f*(*t*) (which is described for each case in the text or figure legends) for a series of randomly generated time-varying circuit input streams **u**(*t*) of length up to 1 s. Up to 200 such time-varying input streams **u**(*t*) were used for training, amounting to at most 200 s of simulated biological time for training the readouts.

The performance of trained readouts was evaluated by measuring the correlation between **w** · **x**(*t*) and the target function *f*(*t*) during separate testing episodes where the circuit received new input streams **u**(*t*) (that were generated by the same random process as the training inputs).

All simulations were carried out with the software package CSIM [61], which is freely available from http://www.lsm.tugraz.at. It uses a C^++^-kernel with Matlab interfaces for input generation and data analysis. As simulation time step, we chose 0.5 ms.

#### Technical details of Figure 5.

Four randomly generated test input streams, each consisting of eight spike trains (see Figure 5A), were injected into four disjoint (but interconnected) subsets of 5 × 5 × 5 = 125 neurons in the circuit consisting of 600 neurons. Feedbacks from readouts were injected into the remaining 100 neurons of the circuit. The set of 100 neurons for which the firing activity is shown in Figure 5C contained 20 neurons from each of the resulting five subsets of the circuit.

*Generation of input streams for training and testing.* The time-varying firing rate *r_i_*(*t*) of the eight Poisson spike trains that represented input stream *i* was chosen as follows. The baseline firing rate for streams 1 and 2 (see the lower half of Figure 5A) was chosen to be 5 Hz, with randomly distributed bursts of 120 Hz for 50 ms. The rates for the Poisson processes that generated the spike trains for input streams 3 and 4 were periodically drawn randomly from the two options 30 Hz and 90 Hz. The actual firing rates (i.e., spike counts within a 30-ms window) resulting from this procedure are plotted in Figure 5B.

To demonstrate that readouts that send feedback into the circuit can just as well represent neurons *within* the circuit, we had chosen the readout neurons that send feedback to be I&F neurons with noise, like the other neurons in the circuit. Each of them received synaptic inputs from a slightly different randomly chosen subset of neurons within the circuit. Furthermore, the signs of weights of these synaptic connections were restricted to be positive (negative) for excitatory (inhibitory) presynaptic neurons.

The eight readout neurons that provided feedback were trained to represent in their firing activity at any time the information in which of input streams 1 or 2 a burst had most recently occurred. If it occurred most recently in input stream 1, they were trained to fire at 40 Hz, and they were trained not to fire whenever a burst had occurred most recently in input stream 2. The training time was 200 s (of simulated biological time). After training, their output was correct 86% of the time (average over 50 s of input streams, counting the high-dimensional attractor as being in the on state if the average firing rate of the eight readout neurons was above 34 Hz). It was possible to train these readout neurons to acquire such persistent firing behavior, although they only received input from a circuit with fading memory, because they were actually trained to acquire the following behavior: fire whenever the rate in input stream 1 becomes higher than 30 Hz, or if one can detect in the current state **x**(*t*) of the circuit traces of recent high feedback values, provided the rate of input stream 2 stayed below 30 Hz. Obviously this definition of the learning target for readout neurons only requires a fading memory of the circuit.

The readouts for the other three tasks achieved in 50 tests for new inputs over 1 s (that had been generated by the same distribution as the training inputs, see the preceding description) showed the following average performance: task of panel E: mean correlation: 0.85, task of panel F: mean correlation: 0.63, task of panel G: mean correlation: 0.86.

#### Technical details of Figure 2.

The same circuit as for Figure 5 was used. First, two linear readouts with feedback were simultaneously trained to become highly active after the occurrence of the cue in the spike input, and then to linearly reduce their activity, but each within a different timespan (400 ms versus 600 ms). Their feedback into the circuit consisted of two time-varying analog values (representing time-varying firing rates of two populations of neurons), which were both injected (with randomly chosen amplitudes) into the same subset of 350 neurons in the circuit. Their weights **w** were trained by linear regression for a total training time of 120 s (of simulated biological time), consisting of 120 runs of length 1 s with randomly generated input cues (a burst at 200 Hz for 50 ms) and noise inputs (five spike trains at 10 Hz).

#### Technical details of Figure 3.

Time-varying firing rates for the two input streams (each consisting of eight Poisson spike trains) were drawn randomly from values between 10 Hz and 90 Hz. The 16 spike trains from the two input streams, as well as feedback from trained readouts were injected into randomly chosen subsets of neurons. In contrast to the experiment for Figure 3, these circuit inputs were not injected into spatially concentrated clusters of neurons, but to a sparsely distributed subset of neurons scattered throughout the three-dimensional circuit. As a consequence, the firing activity *CA*(*t*) of the high-dimensional attractor (see Figure 3D) cannot be readily detected from the spike raster in Figure 3C. Both the linear readout that sends feedback, and subsequently the other two linear readouts (whose output for a test input to the circuit is shown in Figure 3E and 3F), were trained by linear regression during 140 s of simulated biological time.

Average performance of linear readouts on 100 new test inputs of length 700 ms (that had been generated from the same distribution as the training inputs) was—task of panel D, mean correlation: 0.82; task of panel E, mean correlation: 0.71; task of panel F, mean correlation: 0.79.

Control experiments (see Figure 7) show that the feedback is essential for the performance of the circuit for these computational tasks.

**Figure 7:**
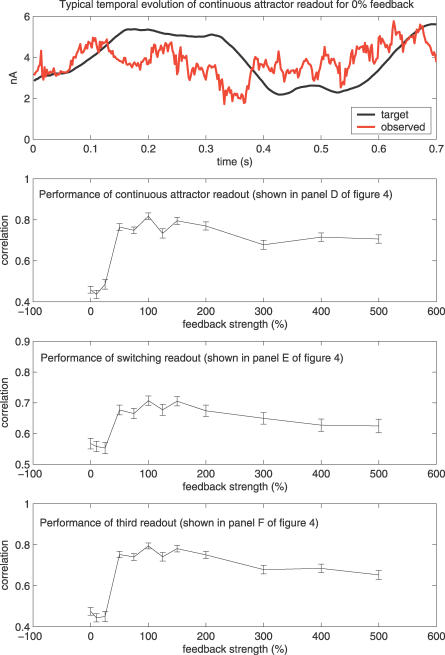
Evaluation of the Dependence of the Performance of the Circuit in Figure 4 on the Feedback Strength (i.e., on the Mean Amplitude of Current Injection from the Readout Back into Neurons in the Circuit) For each feedback strength that was evaluated, the readouts were trained and tested for this feedback strength as for the preceding experiments. Error bars in (B–D) denote standard error. These control experiments show that the feedback is essential for the performance of the circuit.

## Abbreviations

FSM: finite state machine
HH: Hodgkin–Huxley
I&F: integrate and fire
